# Photonic force optical coherence elastography for three-dimensional mechanical microscopy

**DOI:** 10.1038/s41467-018-04357-8

**Published:** 2018-05-25

**Authors:** Nichaluk Leartprapun, Rishyashring R. Iyer, Gavrielle R. Untracht, Jeffrey A. Mulligan, Steven G. Adie

**Affiliations:** 1 Meinig School of Biomedical Engineering, Cornell University, Ithaca, NY 14853 USA; 2 School of Electrical and Computer Engineering, Cornell University, Ithaca, NY 14853 USA; 3 Present Address: Optical and Biomedical Engineering Laboratory, School of Electrical, Electronic and Computer Engineering, The University of Western Australia, Perth, WA 6009 Australia

## Abstract

Optical tweezers are an invaluable tool for non-contact trapping and micro-manipulation, but their ability to facilitate high-throughput volumetric microrheology of biological samples for mechanobiology research is limited by the precise alignment associated with the excitation and detection of individual bead oscillations. In contrast, radiation pressure from a low-numerical aperture optical beam can apply transversely localized force over an extended depth range. Here we present photonic force optical coherence elastography (PF-OCE), leveraging phase-sensitive interferometric detection to track sub-nanometer oscillations of beads, embedded in viscoelastic hydrogels, induced by modulated radiation pressure. Since the displacements caused by ultra-low radiation-pressure force are typically obscured by absorption-mediated thermal effects, mechanical responses of the beads were isolated after independent measurement and decoupling of the photothermal response of the hydrogels. Volumetric imaging of bead mechanical responses in hydrogels with different agarose concentrations by PF-OCE was consistent with bulk mechanical characterization of the hydrogels by shear rheometry.

## Abstract (web summary)

Optical tweezers, while well suited for micro-manipulation, are difficult to apply to volumetric microrheology. Here, Leartprapun et al. combine low-NA optical radiation-pressure forces with sensitive interferometric detection to enable volumetric microrheology with promising applications in biological systems.

## Introduction

Optical manipulation has had a revolutionary impact in the biological and nanoscale sciences^1–3^. Developed by Ashkin et al.^4^, optical tweezers (OTs) have enabled the manipulation of biological systems at the molecular-to-cellular scale. This has led to many seminal studies, including measurement of the elastic properties of bacterial flagella^5^, direct observation of the movement and forces generated by molecular motors^6,7^, the study of mechanotransduction pathways in living cells^8^, and measurement of the mechanical properties and biophysical interactions of DNA^9,10^. OTs utilize the gradient force of a high-numerical aperture (NA) laser beam to achieve trapping and manipulation of micrometer-sized particles near the beam focus. Typical forces that can be achieved range from femtonewtons to hundreds of piconewtons, which covers the range of forces associated with the biophysical processes of life at the molecular-to-cellular scale.

More than a decade before the invention of OTs, Ashkin^11^ demonstrated that low-NA laser beams could exert sufficient radiation-pressure forces to accelerate micrometer-scale dielectric particles along the beam path, or could be used to form a dual-beam trap based on counter-propagating beams. Guck et al.^13^ adopted Ashkin’s original dual-beam trapping configuration to develop the optical stretcher for the study of cell mechanics^12^, and used it to study the mechanical properties of cancer cells and human blood cells^14^. Radiation pressure has been utilized on other microfluidic platforms for nanoparticle sorting and chromatography^15,16^. Compared to OTs, however, optical manipulation based on radiation pressure has led to fewer applications in the life sciences.

The rapidly growing field of mechanobiology^17,18^ has created new opportunities for application of optical manipulation across the micrometer-to-millimeter scale. Over the last decade, mechanobiology research has uncovered the integral role that extracellular matrix (ECM) mechanics and biophysical cell–ECM interactions play in biological processes, including the onset and progression of cancer^19–22^, stem cell differentiation^23–25^, morphogenesis^26^, and wound healing^25^. Biophysical interactions play an integral role across all spatial scales, from molecular processes at the nanoscale^6–10^, to collective (emergent) behavior at the micro- to mesoscale^27–29^. At the micro- to mesoscale, cellular behavior is known to be different in three-dimensional (3D) versus two-dimensional (2D) environments^30,31^. Consequently, there is an important trend toward the adoption of 3D cell culture systems^22,30,31^ that is driving the need for new imaging approaches that can support volumetric imaging of microscale spatial variations in ECM mechanics across time. Atomic force microscopy has been the preferred method in mechanobiology, but it can only interrogate the sample surface. Laser tweezer-based active microrheology^32,33^ (AMR) is a promising approach that utilizes OTs to induce and detect nanometer-scale displacements from micro-beads randomly distributed within 3D cell culture. However, its volumetric throughput is limited by the need for precise 3D alignment of high-NA trapping and position detection beams to each probing bead, prior to actuation with a transversely oscillating optical trap. Although simultaneous trapping and detection of multiple beads can be achieved with time-shared OTs^34^ or holographic OTs^35,36^, multiplexed manipulation of beads randomly distributed over a depth range of a hundred micrometers or greater with OTs has not been demonstrated. Other emerging approaches include Brillouin microscopy^37,38^ and optical coherence elastography^39–41^ (OCE). However, none of the above methods have demonstrated the ability to support mechanical microscopy with both 3D cellular-level resolution and sufficient throughput to facilitate time-lapse imaging studies over millimeter-scale volumes in mechanobiology.

In order to address the unmet need for volumetric, time-lapse mechanical microscopy of engineered systems in mechanobiology research, we revisited Ashkin’s original idea of using radiation pressure from a low-NA beam^11^—now as a potential mechanism to apply localized mechanical excitation to micro-beads embedded in aqueous biological media. The use of low-NA radiation pressure to apply transversely localized axial force over an extended depth range can be advantageous for volumetric data acquisition, but comes with the challenges that a low-NA beam exerts significantly lower force than high-NA OTs, and that historically, studies on isolating the effects of photonic radiation pressure have been hindered by accompanying photothermal responses^11,42,43^. OCT has been used previously to monitor the radiation-pressure-induced trajectories of beads in liquid media^44^; but this method has yet to be applied to solid viscoelastic materials.

In this paper, we present photonic force OCE (PF-OCE) as a technique for 3D mechanical microscopy, leveraging the interferometric displacement sensitivity of OCT to detect picometer-to-nanometer bead oscillations induced by modulated radiation pressure from a low-NA beam. We address the challenge of isolating radiation-pressure effects within absorbing aqueous media via a linear model to decouple mechanical and photothermal responses, combined with a differential scattering approach. PF-OCE has the potential to provide a new platform for large-scale volumetric mechanical microscopy—probing micrometer-scale spatial variations in the mechanical responses of the medium, with a spatial sampling that is statistically controlled by the distribution of the beads inside the medium. Such a capability may readily find applications in cell mechanics and mechanobiology research, for instance, by enabling the mapping of spatio-temporal variations in ECM mechanics for 3D traction force microscopy (TFM)^27,29,31,45^.

## Results

### Principles of PF-OCE

Based on mechanical excitation via harmonically modulated radiation-pressure force from a low-NA beam (hereafter referred to as the PF forcing beam), PF-OCE measures the resulting oscillations of beads embedded in viscoelastic media induced by the PF forcing beam (the mechanical response) by compensating for the accompanying absorption-mediated photothermal effects of the aqueous medium (the photothermal response).

We begin with the theoretical basis for using harmonically modulated radiation pressure to induce oscillations of beads embedded in a viscoelastic medium. Generalizing Ashkin’s simplified expression^11^ to include the contribution of both photon scattering and absorption to the net change in linear momentum, the axial radiation-pressure force, **F**_rad_, exerted on a neutral particle by a weakly focused beam with optical power *P*, is given by:

1 $${\mathbf{F}}_{{\mathrm{rad}}} = \frac{{(2q_{\mathrm{s}} + q_{\mathrm{a}})n_{{\mathrm{med}}}P}}{c}{\hat{\mathbf z}},$$

where *c* denotes the speed of light in vacuum, *n*_med_ denotes refractive index of the medium, and ${\hat{\mathbf z}}$ denotes a unit vector pointing in the propagation direction of the forcing beam. The proportionality constants *q*_a_ and *q*_s_ define the fractions of incident photon momentum that are imparted to the bead in the direction ${\hat{\mathbf z}}$ as a result of absorption and scattering, respectively. For a non-absorbing particle, such as the latex (polystyrene) beads used in Ashkin’s experiments, the contribution of *q*_a_ to the radiation-pressure force is neglected. The proportionality constant *q*_s_ accounts for the effects of shape, size, and scattering cross section of the bead in relation to the characteristics of the forcing beam (for example, wavelength and beam waist radius or NA), the refractive indices of the bead and medium, and the position of the bead in 3D space relative to the forcing beam.

Together with the restoring force from the viscoelastic medium, harmonically modulated **F**_rad_ induces oscillatory motion of the bead. Oestreicher provided a theoretical model for the impedance of an oscillating sphere in a linear viscoelastic medium^46^. We inverted equation (18) in Oestreicher’s paper^46^ to obtain an expression for the oscillation amplitude of the bead as a function of bead radius, *a*, complex shear modulus, $G^\ast$(*ω*) = *G*′(*ω*) + *iG*″(*ω*), and mass density, *ρ*, of the medium, given by

2 $$u_0(\omega ) = \frac{{F_{{\mathrm{rad}}}}}{{6\pi a\left| {G_{{\mathrm{eff}}}(\omega )} \right|}},$$

where

3 $$G_{{\mathrm{eff}}}(\omega ) = \frac{{\rho a^2\omega ^2}}{9} - G^ \ast (\omega )\left[ {1 - i\sqrt {\frac{{\rho a^2\omega ^2}}{{G^ \ast (\omega )}}} } \right].$$

In Eq. (2), *u*_0_ describes the oscillation amplitude of the bead resulting from harmonically modulated axial radiation-pressure force with peak magnitude $F_{{\mathrm{rad}}} = \left\| {{\mathbf{F}}_{{\mathrm{rad}}}} \right\|$ and modulation (angular) frequency *ω*. We observe that *u*_0_ is directly proportional to the magnitude of axial radiation-pressure force but inversely proportional to higher-order powers of the bead radius.

For non-absorbing beads embedded in an aqueous medium, the scattering-mediated radiation pressure exerted on the bead from the PF forcing beam is accompanied by the absorption-mediated photothermal response of the medium. Absorption-mediated responses form the basis of multiple functional imaging modalities. Photoacoustic tomography uses short laser pulses to generate ultrasonic pressure waves from absorption-induced thermoelastic expansion^47^. High-power laser pulses have also been used to generate propagating surface acoustic waves caused by thermal expansion for elastography applications^48^. Absorption-induced optical path length (OPL) change, governed by the thermo-optic effect and thermal expansion, allows photothermal OCT (PT-OCT) to detect the presence of chromophores in biological samples^49,50^.

In order to understand and account for the effects of absorption on OPL, consider the case of a non-absorbing dielectric bead embedded at depth *L* in a homogeneous absorbing medium with uniform (spatially invariant) refractive index *n*_med_. The OPL to the bead measured by OCT is encoded in the phase of the complex OCT signal, given by *Φ* = (4π/*λ*) ⋅ *n*_med_*L*. The OPL to the bead corresponds to the product OPL = *n*_med_L. Both *n*_med_ and *L* can vary with a change in temperature via two different phenomena—*n*_med_ via the thermo-optic effect and *L* via thermal expansion. The OPL change with respect to the change in temperature can be expressed via the product rule of differentiation as,

4 $$\frac{{{\mathrm{dOPL}}}}{{{\mathrm{d}}T}} = \frac{{{\mathrm{d}}n_{{\mathrm{med}}}}}{{{\mathrm{d}}T}}L(T) + n_{{\mathrm{med}}}(T)\frac{{{\mathrm{d}}L}}{{{\mathrm{d}}T}}.$$

The derivatives d*n*_med_/d*T* and d*L*/d*T* are the thermo-optic coefficient and the thermal expansion coefficient of the medium, respectively. Equation (4) describes how the measured OPL change due to the change in temperature of the medium originates from both the change in refractive index and thermal expansion, and does not directly correspond to physical displacement of the bead.

Assuming that scattering and absorption are independent events within the context of PF-OCE, we model the measured bead OPL oscillation (the total response), ΔOPL_tot_, due to the modulated PF forcing beam as a linear combination of the complex mechanical response of the bead, ΔOPL_mech_, and the complex photothermal response of the medium, ΔOPL_PT_, given by

5 $$\Delta {\mathrm{OPL}}_{{\mathrm{tot}}}({\mathbf{r}},t,\omega ) = {\mathrm{\Delta OPL}}_{{\mathrm{mech}}}({\mathbf{r}},t,\omega ) + {\mathrm{\Delta OPL}}_{{\mathrm{PT}}}({\mathbf{r}},t,\omega )$$

where ΔOPL_mech_ and ΔOPL_PT_ are given by

6 $${\mathrm{\Delta OPL}}_{{\mathrm{mech}}}({\mathbf{r}},t,\omega ) = A_{{\mathrm{mech}}}({\mathbf{r}},\omega )e^{i(\omega t + \varphi _{{\mathrm{drive}}} + \varphi _{{\mathrm{mech}}}({\mathbf{r}},\omega ))}$$

and

7 $${\mathrm{\Delta OPL}}_{{\mathrm{PT}}}({\mathbf{r}},t,\omega ) = A_{{\mathrm{PT}}}({\mathbf{r}},\omega )e^{i(\omega t + \varphi _{{\mathrm{drive}}} + \varphi _{{\mathrm{PT}}}({\mathbf{r}},\omega ))}.$$

**Fig. 1:**
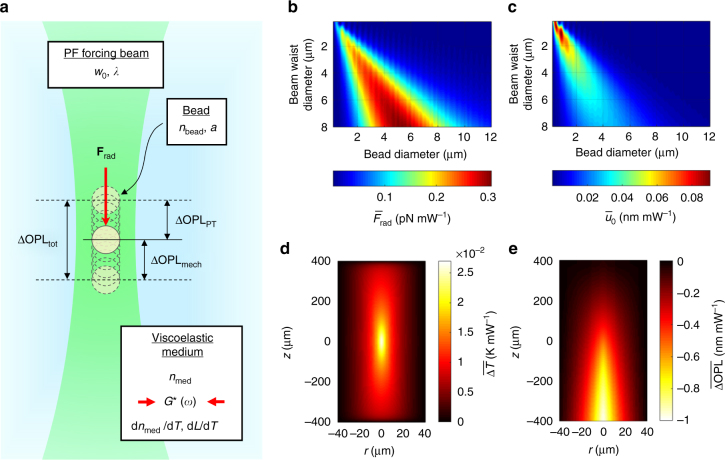
Underlying principle of PF-OCE and theoretical prediction of bead responses. **a** Cartoon illustration of the working principle of PF-OCE depicting mechanical excitation by modulated radiation-pressure force, **F**_rad_, from a low-NA beam and various factors, associated with the PF forcing beam, the bead, and the viscoelastic medium, that affect the measurement of change in OPL. **b** Magnitude of normalized forward radiation-pressure force, $\bar F_{{\mathrm{rad}}}$, from a Gaussian beam (*λ* = 976 nm) on a non-absorbing spherical bead embedded in a medium (refractive indices *n*_bead_ = 1.58 and *n*_med_ = 1.34) as a function of bead diameter, 2*a*, and beam waist diameter, 2*w*_0_, obtained from simulation based on generalized Lorenz-Mie theory (GLMT). **c** Normalized (physical) bead oscillation amplitude, $\bar u_0$, resulting from harmonically modulated radiation-pressure force with peak amplitude $\bar F_{{\mathrm{rad}}}$ and modulation frequency *ω* = 2π (20 Hz) for a viscoelastic medium with G*(*ω*) = 250 + 4*i* (Pa). **d** Map of normalized change in temperature, $\overline {\Delta T}$, of an aqueous medium after 50 ms of exposure to a Gaussian beam (*λ* = 976 nm, *w*_0_ = 3.19 μm). **e** Map of cumulative optical path length change, $\overline {{\mathrm{\Delta OPL}}}$, induced by $\overline {\Delta T}$ based on a PT-OCT model (Supplementary Methods). Note that (**e**) represents OPL and not physical distance. In (**d**) and (**e**), *r* and *z* denote the radial and axial (depth) coordinates, defined w.r.t. the focus of the beam, respectively

The vector **r** = (*x*, *y*, *z*) denotes the spatial coordinates of each pixel in the OCT image and *φ*_drive_ denotes the phase of the PF forcing beam drive waveform at time *t* = 0. *A*_mech_ and *φ*_mech_ denote the amplitude and phase of the complex mechanical response, respectively. Likewise, *A*_PT_ and *φ*_PT_ denote the amplitude and phase of the complex photothermal response, respectively. The goal of PF-OCE is to isolate the complex mechanical response of the bead, which is dependent on the mechanical properties of the surrounding viscoelastic medium, from the measured total response by subtracting the accompanying photothermal response of the medium (Fig. 1a).

### Theoretical simulations

In order to understand the effects of each design parameter (for example, NA of the PF forcing beam and bead size) on the measured response, and to obtain an estimate of the expected magnitude of the mechanical and photothermal responses, we simulated the contributions of both radiation-pressure force and photothermal response to the total OPL oscillation of a polystyrene bead. Unless stated otherwise, all numerical results presented in this section were obtained from theoretical simulation assuming a Gaussian PF forcing beam with wavelength *λ* = 976 nm and waist radius *w*_0_ = 3.19 μm and a spherical bead with refractive index *n*_bead_ = 1.58. Refractive index of the medium was assumed to be *n*_med_ = 1.34 for biological hydrogels.

An accurate estimate of the scattered (and absorbed) photon energy is critical to predict the magnitude of radiation-pressure force on a bead. Several approaches have been used to estimate *q*_s_ and *q*_a_ in Eq. (1). Among them, Generalized Lorenz-Mie Theory (GLMT)^51^ is applicable for estimating *F*_rad_ from a focused Gaussian beam on a bead of arbitrary shape and size. Our MATLAB implementation of GLMT showed that the normalized axial radiation-pressure force (that is, *F*_rad_ per unit power), $\bar F_{{\mathrm{rad}}}$, from the PF forcing beam on a bead was on the order of 0.2–0.3 pN mW^−1^ at the focal plane when the focal spot size of the beam was comparable to the bead diameter (Fig. 1b). The force was lower as the focal spot size deviated from the bead diameter. Moreover, $\bar F_{{\mathrm{rad}}}$ increased monotonically with both beam waist and bead diameter when the two dimensions were comparable, whereas $\bar u_0$ decreased beyond a certain point (Fig. 1c) because the oscillation amplitude is both directly proportional to $\bar F_{{\mathrm{rad}}}$ and inversely proportional to *a* (Eqs. (2) and (3)).

In order to simulate the photothermal response, we solved the heat transfer equation to estimate the change in temperature of the medium due to absorption and then modified a theoretical model given for PT-OCT by Lapierre et al.^50^ to estimate the resulting cumulative OPL change for a general case with spatially varying *n*_med_ and *T*. Detailed descriptions of the theoretical model and parameters used for the simulation can be found in the Supplementary Methods and Supplementary Table 1. In an aqueous medium, the normalized change in temperature per unit power, $\overline {\Delta T}$, due to absorption by water molecules was simulated to be on the order of 10^−2^ K mW^−1^ after 50 ms of continuous exposure to the PF forcing beam (Fig. 1d). The resulting normalized cumulative OPL change, $\overline {\Delta {\mathrm{OPL}}}$, due to photothermal effects was on the order of 0.5 nm mW^−1^ (Fig. 1e), approximately an order of magnitude larger than $\bar u_0$ measured for the same beam parameters (*λ* = 976 nm, *w*_0_ = 3.19 μm, and *n*_med_ = 1.34; Fig. 1c, e).

These simulations assume that the OPL change induced by the photothermal effects of a PF forcing beam on the medium is unaffected by the presence or size of the beads. On the other hand, both the radiation-pressure force and the resulting bead oscillation amplitude for a given medium and PF forcing beam can vary by an order of magnitude depending on the size of the bead alone. This provides a guide for designing experimental conditions that affect the mechanical response without disturbing the photothermal response, forming the basis for the isolation of bead mechanical response from the measured total response (see Isolation of bead mechanical response).

### Experimental setup and data acquisition

**Fig. 2:**
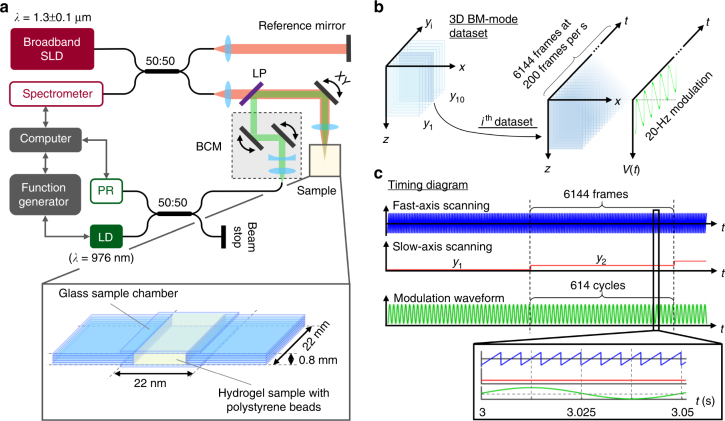
Optical setup and data acquisition scheme for volumetric mechanical microscopy with PF-OCE. **a** The optical setup consisted of an SD-OCT system and a PF forcing beam combined in free space with the OCT sample arm beam. A function generator provided external modulation of the power of the PF forcing beam. **b** Illustrations of 3D BM-mode dataset for the beam-scanning (200-Hz frame rate) and modulation waveform (20-Hz modulation frequency) from the function generator for a 3D BM-mode acquisition. **c** Associated timing diagram for the acquisition scheme. SLD: superluminescent diode, LD: laser diode, PR: photoreceiver, LP: long-pass dichroic filter, BCM beam control module, *XY* two-axis galvanometer

To enable simultaneous mechanical excitation by radiation pressure from the PF forcing beam and detection of resulting OPL oscillations by phase-sensitive OCT, we combined the PF forcing beam (*λ* = 976 nm) with the sample arm beam of a spectral-domain (SD)-OCT system via a free-space beam control module (BCM) and a dichroic filter (Fig. 2a). In this configuration, both the OCT and the PF forcing beams were collinearly scanned in a raster pattern by the same galvanometer and focused by the objective lens to the same position in 3D space. The waist radius of the PF forcing beam was measured to be 3.19 µm (NA = 0.1) at the focal plane (Supplementary Fig. 1 and Supplementary Methods). Although the theoretical simulation suggests that a PF forcing beam with smaller waist radius (when paired with comparable bead size) is optimal for maximizing the bead oscillation amplitude (Fig. 1b), we chose not to increase the NA of the PF forcing beam beyond 0.1 to ensure that radiation-pressure force would be applied over an extended depth range for high-throughput volumetric measurement.

In this paper, we used agarose hydrogels of different concentrations confined in a glass chamber as examples of viscoelastic substrates used in cell imaging applications (Fig. 2a). Polystyrene beads with mean diameter of 3 µm were added to the hydrogels to serve as scattering particles for oscillation by radiation pressure. With a typical shear modulus in the range of 0.1–1 kPa^52^, we expected $\bar u_0$ to be approximately 0.03 nm mW^−1^. Agarose hydrogels of uniform concentrations were used to validate PF-OCE with shear rheometry, and a side-by-side sample of two agarose concentrations on either side of the sample chamber was used to demonstrate the volumetric capabilities of PF-OCE. To enable 3D volumetric measurements of OPL oscillations of the 3 µm beads, we adopted a 3D BM-mode acquisition scheme, wherein the beams were repeatedly raster scanned to acquire multiple B-scans at each slow-axis location (Fig. 2b, c). This scanning configuration allows continuous beam-scanning acquisition along the fast axis (B-scan) while also supporting OPL tracking at each spatial location over time (M-scan) (refer to Methods for further details on the acquisition scheme).

The BM-mode acquisition scheme has two key implications for the implementation of PF-OCE in biological systems typically used for live-cell-imaging studies. First, the fast-axis beam-scanning effectively resulted in a pulse-train mechanical excitation on each of the 3-µm beads instead of a continuous sinusoidal waveform provided by the function generator (Supplementary Fig. 2). As a B-scan was acquired, the PF forcing beam would dwell on each of the 3-μm beads for approximately 67 μs (corresponding to four A-scans), after which the bead received no force until the PF forcing beam scanned over the bead again in the next frame. In other words, the actual excitation on each bead is a frequency comb with a 20-Hz fundamental frequency and additional higher-order harmonics. This implies that the frequency-dependent response of the medium must be accounted for when quantitatively reconstructing absolute mechanical properties of the medium from the bead mechanical responses; this subject will be addressed in a future manuscript. Under this type of excitation, the time-averaged optical power imparted on each 3-μm bead by the PF forcing beam was only 0.3 mW for a peak power of 112 mW, as opposed to 56 mW that would have been expected from a continuous sinusoidal excitation with the same peak power. Although this outcome is expected to result in a lower bead oscillation amplitude compared to the continuous excitation case, the two orders of magnitude reduction in the time-averaged optical power imparted on the sample is beneficial for biological studies where cell viability is a concern.

Second, there is a trade-off between acquisition speed and OPL oscillation measurement sensitivity. In the shot-noise limit, the sensitivity of OPL oscillation amplitude measurement by OCT is approximately inversely proportional to the square-root of the number of BM-mode frames acquired per slow-axis position^53,54^. Prioritizing sensitivity over speed, we acquired up to 6144 frames per slow-axis position at a rate of 200 frames per second and achieved an OPL oscillation amplitude noise floor of 105 pm, approximately 50 pm above the theoretical shot-noise limit (Supplementary Methods), for OCT signals with signal-to-noise ratio (SNR) of 25 dB (Supplementary Fig. 3). This acquisition scheme requires at least 5 min to acquire OPL oscillation data over a transverse field of view (FOV) of 200 μm × 10 μm at a spatial sampling of 1 µm per pixel; this spatial sampling density ensures each 3-µm bead was sampled multiple times along the fast and slow axes. For typical volumes used in biological studies such as the side-by-side sample presented in this paper with a FOV of 200 μm × 125 μm, acquiring 3072 frames at 490 frames per second offers a 5× reduction in the acquisition time to 13 min, at the cost of increasing the OPL oscillation amplitude noise floor to 180 pm. Alternatively, the acquisition time can be shortened without sacrificing the OPL oscillation amplitude sensitivity by increasing the frame rate while maintaining spatial sampling over a large FOV; this can be achieved with a resonant scanner or other high-speed beam-scanning options^55^.

### Isolation of bead mechanical response

Based on the linear model in Eqs. (5)–(7), the mechanical response of the 3-µm beads can be isolated from the measured total response by subtracting the photothermal response of the medium. This approach requires availability of a reliable estimate of the photothermal response. In principle, the complex photothermal response in a uniformly absorbing medium may be theoretically obtained from the model of the absorption-mediated OPL change in PT-OCT^50^. However, we were not able to ascertain the accuracy of the theoretical simulation (Supplementary Methods) under our experimental conditions due to the lack of available material properties for the agarose hydrogels used in our experiments as well as the added contribution of the confined glass chamber in the experimental setup to the heat transfer process and thermal expansion model (Supplementary Discussions). Alternatively, under the premise that weak scattering signals from the medium would produce adequate OCT signals for OPL measurements but would not be sufficient to produce detectable mechanical response induced by scattering-mediated radiation-pressure force, a differential scattering approach could be employed wherein the complex photothermal response may be measured experimentally from weak scatterers in the sample. These weak scatterers, thus, act as reporters of the photothermal response, without producing measurable displacements resulting from photon momentum transfer.

In implementing this differential scattering approach, we leveraged the size-dependence of backscattering intensity^3^ and added 0.1-µm polystyrene beads to the sample to provide weak background scattering signals for measuring the photothermal response. In the Supplementary Methods, we estimate that $\bar u_0$ for the 0.1-µm beads is expected to be four orders of magnitude smaller than $\bar u_0$ for the 3-μm beads, based on their differences in OCT scattering intensity and theoretical predictions.

**Fig. 3:**
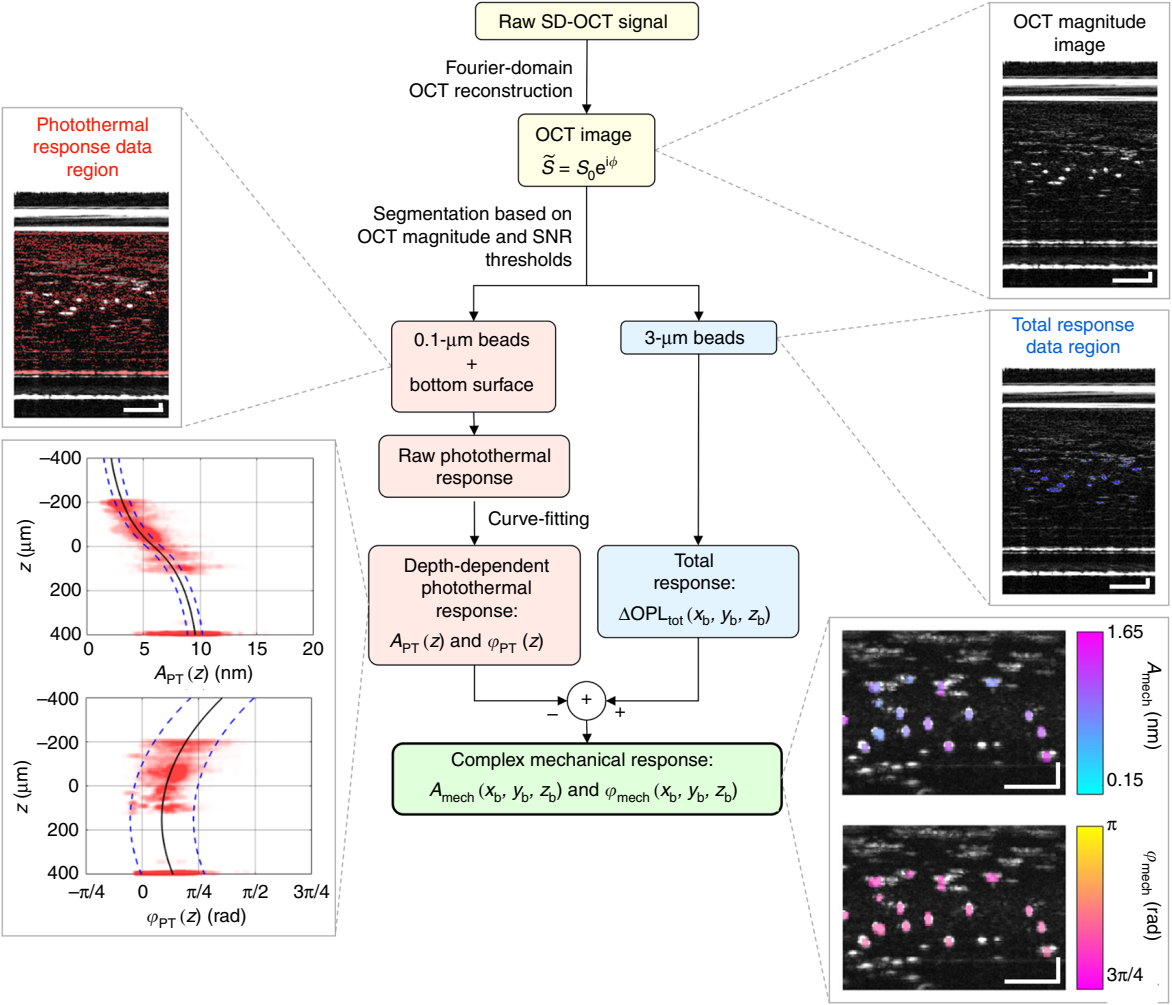
Data-processing flow chart outlining key steps to isolate the mechanical response in PF-OCE. The OCT image was segmented into total response data region (3-μm beads) and photothermal response data region (0.1-μm beads and bottom surface of the sample chamber) with magnitude and SNR thresholds (Supplementary Table 2 provides exact values of the thresholds used in the presented experiments). Magnitude and phase of raw photothermal response data were fit to a theoretical photothermal response curve and a cubic polynomial function, respectively, to obtain depth-dependent photothermal response, given by amplitude *A*_PT_ (*z*) and phase *φ*_PT_ (*z*). The mechanical response of each 3-μm bead, given by amplitude *A*_mech_ (*x*_b_, *y*_b_, *z*_b_) and phase *φ*_mech_ (*x*_b_, *y*_b_, *z*_b_), where (*x*_b_, *y*_b_, *z*_b_) are the set of pixel coordinates corresponding to each 3-μm bead, was isolated after subtracting the photothermal response at corresponding depths from the measured total response. Example images from a 0.4% agarose hydrogel dataset are provided at each key processing step

The process to isolate the mechanical responses of the 3-µm beads is summarized in a flow chart (Fig. 3). In order to obtain a reliable estimate of the photothermal response, it was important to account for the consequences of the low SNR of OCT signals^53,54^ from the weakly scattering 0.1-μm beads. The low OCT SNR (<12 dB) of the 0.1-µm beads resulted in relatively large OCT phase noise on the measured OPL oscillations (that is, the raw photothermal response). To reduce the contribution of individual OCT phase measurement errors, we calculated the depth-dependent photothermal response (with amplitude *A*_PT_ (*z*) and phase *φ*_PT_ (*z*)) by performing curve-fitting of the amplitude and phase of the raw photothermal response as a function of depth (Fig. 3), using the theoretical simulation of the absorption-mediated OPL change^50^ for the amplitude and a cubic polynomial function for the phase. For small FOVs, we assumed that water was the only absorber in the sample and that the water concentration was transversely uniform across the sample, resulting in a photothermal response that is dependent on depth alone. This combined experimental and theoretical approach yielded estimates of the depth-dependent photothermal response from the fitted curves with uncertainties of approximately ±0.7 nm for *A*_PT_ (*z*) and ±0.5 rad for *φ*_PT_ (*z*) (Supplementary Figs 4 and 5). For larger volumes, such as the side-by-side sample presented here, this uncertainty can be maintained or reduced by performing depth-dependent photothermal curve-fitting on smaller localized sub-volumes. The mechanical responses of the 3-µm beads, ΔOPL_mech_, were subsequently isolated from the measured total responses, ΔOPL_tot_, by subtracting the depth-dependent photothermal responses (values obtained from the best-fit curves at the depths of the 3-µm beads) from the total responses.

This differential scattering approach relies on four fundamental assumptions. First, we assumed that absorption events and scattering events that occurred in the sample were independent and separable. This assumption must hold for the linear model in Eqs. (5)–(7) to be valid. Second, we assumed that the mechanical response of the 0.1-μm beads was negligible. This assumption was supported by both theoretical predictions and experimental observation of *F*_rad_^44^, and experimental observation of OCT scattering intensity (Supplementary Methods). Third, we assumed that the photothermal response in our single-concentration samples was transversely uniform within the volume considered for photothermal fitting. Under this assumption, the uncertainty from the curve fits may impose a depth-dependent systematic error that affects the accuracy of the isolated amplitude and phase of the mechanical responses relative to their true values, but does not degrade the precision for distinguishing microscale variations in the mechanical responses within a sample. However, if the photothermal response was to have a transverse variation that was unaccounted for, these uncertainties could also impose a random error that adversely affects the ability of PF-OCE to distinguish relative differences in the mechanical responses within a sample. Lastly, we assumed the photothermal response measured on the 3-μm beads was equivalent to the photothermal response measured on the 0.1-μm beads at the same depth. In other words, we assumed that any perturbations of the photothermal response specifically due to the presence of the 3-μm beads were negligible.

### Comparison to shear rheometry

**Fig. 4:**
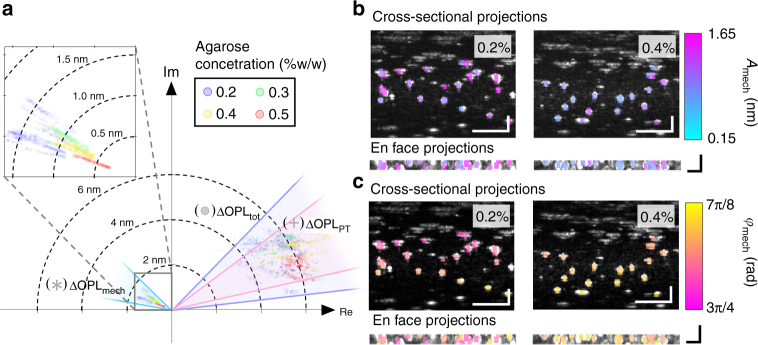
PF-OCE measurements in four agarose hydrogel samples with different agarose concentrations. **a** All measurements of ΔOPL_tot_ (*x*_b_, *y*_b_, *z*_b_) (circle), ΔOPL_PT_ (*z*_b_) (cross) after curve-fitting, and ΔOPL_mech_ (*x*_b_, *y*_b_, *z*_b_) (star) displayed on the complex plane. **b** *A*_mech_ (*x*_b_, *y*_b_, *z*_b_) from one 3D BM-mode dataset in 0.2 and 0.4% agarose samples displayed as a map overlaid on cross-sectional and en face OCT image maximum intensity projections (grayscale). **c** Corresponding maps of *φ*_mech_ (*x*_b_, *y*_b_, *z*_b_) from the same datasets as in **b**. Scale bars: 50 μm (white, cross-sectional projections) and 20 μm (black, en face projections). See Fig. 5 for quantitative comparison to shear rheometry and Supplementary Fig. 10 for cross-sectional and en face projections of *A*_mech_ (*x*_b_, *y*_b_, *z*_b_) and *φ*_mech_ (*x*_b_, *y*_b_, *z*_b_) for all four agarose hydrogel concentrations

The mechanical responses of the 3-µm beads were measured in four agarose hydrogel samples with different mechanical properties and compared to the bulk characterization of the hydrogels by shear rheometry (Supplementary Methods). The complex total responses measured on the 3-µm beads, the fitted photothermal responses at the corresponding depths, and the isolated mechanical responses are displayed on the complex plane (Fig. 4a). We qualitatively observed that ΔOPL_tot_ and ΔOPL_PT_ had comparable magnitudes, and were distributed over the same quadrant of the complex plane for all agarose concentrations. In contrast, ΔOPL_mech_ was approximately four times smaller in magnitude and was in a different quadrant. These observations suggest that the measured total responses were dominated by contributions from photothermal effects, which can be explained by absorption of water molecules in the hydrogel samples at the PF forcing beam wavelength^56^. The fact that ΔOPL_PT_ and ΔOPL_mech_ are in different quadrants of the complex plane implies that the photothermal responses and the mechanical responses do not occur in-phase. Additionally, ΔOPL_mech_ followed a general trend of decreasing magnitude with increasing agarose concentration (Fig. 4a), consistent with our expectation that a higher agarose concentration would produce a stiffer hydrogel. This trend is reflected in Fig. 4b, where the median amplitude of the mechanical response (calculated from a set of spatial pixels corresponding to each 3-µm bead as described in Methods) is overlaid on top of the OCT image of 0.2 and 0.4% agarose samples. Similarly, *φ*_mech_ of 0.4% agarose was closer to π than 0.2% agarose (Fig. 4c). In addition to mechanical contrast between samples, these maps also revealed the variability in *A*_mech_ and *φ*_mech_ of different beads within each sample, which may reflect microscale heterogeneity in the structural and mechanical properties of low-concentration agarose hydrogels^57–59^. For instance, beads with higher *A*_mech_ within a sample could be those inside larger pores, diffusing in the fluid phase of the biphasic porous hydrogel, whereas those with lower *A*_mech_ could be trapped in the solid agarose polymer matrix^57–59^.

**Fig. 5:**
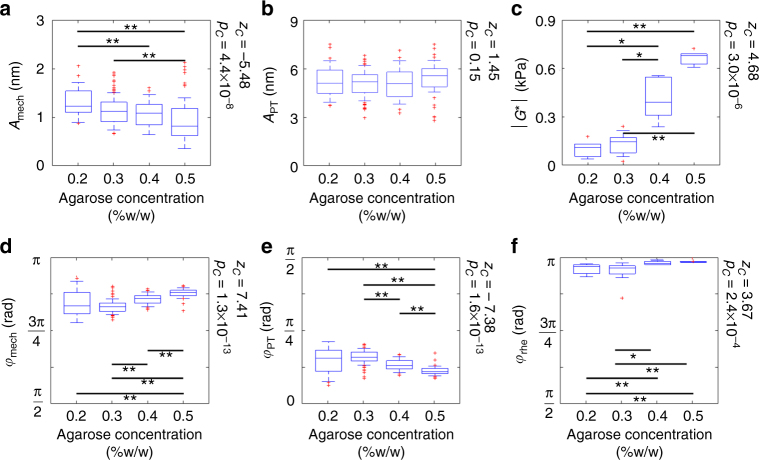
Box plots comparing the PF-OCE responses in agarose hydrogels to bulk mechanical characterization of the hydrogels by shear rheometry. Magnitude and phase of **a**, **d** mechanical response, and **b**, **e** photothermal response compared to **c**, **f** magnitude and phase of the complex shear modulus measured by parallel-plate shear rheometry. Plotted data from PF-OCE measurements represent median values obtained from each of the 3-μm beads within the FOV (total of *N* = 54, 74, 58, and 52 beads for 0.2%, 0.3%, 0.4%, and 0.5% agarose hydrogel samples, respectively). Rheometer measurements were taken on three separate samples, with three repetitions per sample, for each agarose concentration (total of *N* = 5, 9, 9, and 6 independent measurements of 0.2%, 0.3%, 0.4%, and 0.5% agarose hydrogel samples, respectively). Horizontal lines within boxes indicate median values, boxes denote interquartile ranges. Whiskers on the box plots span one standard deviation; data outside of this range are shown in red markers. Black bar and asterisks indicate a statistically significant difference between two agarose concentrations per Kruskal–Wallis test at *p* < 0.05 (*) and *p* < 0.005 (**) confidence levels. *z*_C_ and *p*_C_, respectively, denote normalized test statistic and associated *p*-value for Cuzick’s test for trend across the four agarose concentrations, ordered from 0.2 to 0.5% w/w; thus, *z*_C_ > 0 indicates an increasing trend while *z*_C_ < 0 indicates a decreasing trend. A trend was considered statistically significant if *p*_C_ < 0.05

For quantitative comparisons to standard shear rheometry, *A*_mech_, *A*_PT_, *φ*_mech_, and *φ*_PT_ measured by PF-OCE are displayed as box plots next to the magnitude of complex shear modulus, $|G^\ast |$, and phase delay, $\varphi _{{\mathrm{rhe}}} = \tan ^{ - 1}(G{\prime\prime} {\mathrm{/}}G{\prime} ),$ measured by shear rheometry (Fig. 5). The total response is omitted here but can be found in Supplementary Fig. 6. Complete results from shear rheometry can be found in Supplementary Fig. 7. The mechanical response of the three imaging locations for each concentration of hydrogel can be found in Supplementary Fig. 8. We found a statistically significant (*p*_C_ < 0.05, see Methods for details) monotonically decreasing trend in *A*_mech_ versus agarose concentration (Fig. 5a). This behavior agrees with progressively increasing $|G^\ast |$ of the hydrogels as the agarose concentration increased (Fig. 5c). In contrast, no significant trend (*p*_C_ = 0.15) or difference across agarose concentrations was observed for *A*_PT_ (Fig. 5b). We also found a similar increasing trend (*p*_C_ < 0.05) in both *φ*_mech_ and *φ*_rhe_ toward π as the agarose concentration increased (Fig. 5d, f). The phase delay approaching π is consistent with the response of a predominantly elastic material excited above its damped natural frequency. In contrast, *φ*_PT_ followed an opposite trend (*p*_C_ < 0.05) and was closer to 0 for all hydrogels (Fig. 5e). We note that *φ*_mech_ was up to π/4 rad smaller than *φ*_rhe_ for all concentrations. The discrepancies may reflect the differences between bulk responses of the hydrogels measured by shear rheometry and microscale mechanical responses measured by PF-OCE. Nevertheless, both rheometry and PF-OCE suggest that the hydrogels become more elastic as the agarose concentration increases, which is consistent with previous studies that reported decreases in porosity and influence of viscous drag (due to fluid flow through pores) at higher agarose concentrations^58,59^. Our results demonstrate that the isolated complex mechanical responses of the 3-µm beads from PF-OCE can be used to distinguish different viscoelastic properties of agarose hydrogels, whereas no significant trend that directly correlates to the mechanical properties was observed in the photothermal responses.

### Volumetric mechanical microscopy of agarose hydrogels

**Fig. 6:**
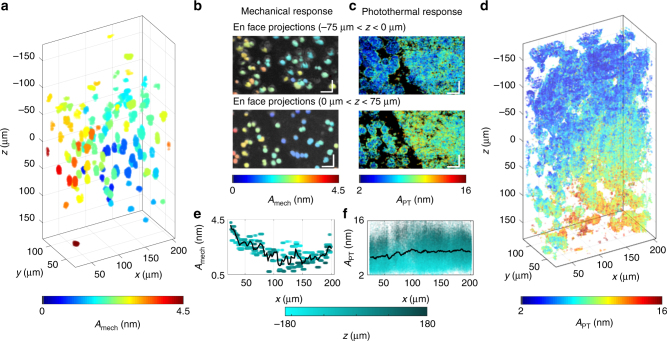
PF-OCE measurements in the side-by-side sample in the vicinity of the boundary. **a** *A*_mech_ (*x*_b_, *y*_b_, *z*_b_) in 3D space (*N* = 220 beads). **b** Two en face projections of *A*_mech_ (*x*_b_, *y*_b_, *z*_b_) over a 75 μm range above and 75 μm range below the plane *z* = 0 μm, overlaid on the OCT image. **c** Two en face projections of *A*_PT_ (*x*, *y*, *z*) corresponding to 0.1-μm beads over a 75 μm range above and 75 μm range below the plane *z* = 0 μm, overlaid on the OCT image. **d** *A*_PT_ (*x*, *y*, *z*) in 3D space. **e**, **f** 2D plot of mechanical and photothermal response against its transverse location **e** (*A*_mech_ (*x*_b_)) and **f** (*A*_PT_ (*x*)), shaded according to their axial positions. The black line shows the mean of all *A*_mech_ (*x*_b_, *y*_b_, *z*_b_) and *A*_PT_ (*x*, *y*, *z*) at each location on the *x*-axis. (Scale bars: 20 μm. See Supplementary Movie 1, which sweeps through responses of en face and cross-sectional projections, in addition to showing the OCT images.)

To demonstrate the volumetric imaging capabilities of PF-OCE, the mechanical response of the beads embedded in the side-by-side sample (0.2% agarose hydrogel on one side and 1% agarose hydrogel on the other) was measured. The sample was aligned such that the boundary was roughly located at the center of the fast-axis (*x*-axis) scanning field, with 0.2% agarose hydrogel on the left half and the 1% agarose hydrogel on the right. Figure 6a shows the 3D distribution of *A*_mech_ (*x*_b_, *y*_b_, *z*_b_), which reveals a clear contrast between the two halves of the sample. This is particularly pronounced for the 3-μm beads between 0 μm < *z* < 75 μm, where the beads on the left (of the *x*-axis) mostly have larger *A*_mech_ (*x*_b_, *y*_b_, *z*_b_) (shaded yellow to red) compared to the those on the right (shaded blue; Fig. 6b). Notably, a sharp boundary is also apparent in the en face projection of the photothermal responses at these depths due to the drastic increase in the number of the low-intensity photothermal reporters on the right side (Fig. 6c). This can be attributed to the intrinsic scattering (in addition to the exogenous 0.1-µm beads added) in the 1% agarose hydrogel, which is present to a significantly higher extent than in the lower-concentration 0.2% counterpart. Thus, the contrast in the bead mechanical responses in Fig. 6b is consistent with the sharp boundary between the two agarose concentrations revealed by the intrinsic background scattering in Fig. 6c. In contrast, the concentration of low-intensity photothermal reporters appears more uniform on each of the sides for depths −75 μm < *z* < 0 μm (Fig. 6c). The spatial variations of *A*_mech_ (*x*_b_, *y*_b_, *z*_b_) at these depths similarly show a more gradual decrease from the softer 0.2% agarose hydrogel to the stiffer 1% agarose hydrogel (Fig. 6b). We speculate that this could indicate a larger extent of interaction (water exchange^57–59^) between the two concentrations at these depths. Overall, we observe that *A*_mech_ (*x*_b_, *y*_b_, *z*_b_) of the beads embedded in the 0.2% hydrogel is approximately 2 nm larger than those in the 1% hydrogel on average, confirming that the 0.2% hydrogel is softer (Fig. 6a, e). Additionally, the photothermal response of the 0.1-µm beads appears transversely homogenous and increases with depth in accordance with the theoretical trends (Fig. 6d, f).

In addition to the expected contrast between the two sides, the 3D distribution of 3-μm beads in Fig. 6a also highlights the local variations in *A*_mech_ (*x*_b_, *y*_b_, *z*_b_) similar to those in the single-concentration datasets (Fig. 4b, c), which could arise due to fluid transfer between the two sides, or the heterogeneous nature of hydrogels. Comprehensive discussions of sources of variability in PF-OCE measurements can be found in Supplementary Discussions. We note that *A*_mech_ (*x*_b_, *y*_b_, *z*_b_) in the 0.2% agarose hydrogel in this dataset is higher than in the single-concentration dataset (Figs. 4b and 5a). This could be attributed to improvements made to the beam profile of the PF forcing beam as the BCM was re-adjusted at the beginning of the side-by-side experiment. Finally, with the current acquisition settings, the 3-µm beads (*N* = 220 beads in total) analyzed in this 3D volume were acquired in 13 min resulting in an average acquisition time of 3.55 s per bead, which represents a greater than 2× speed improvement compared to state-of-the-art OT-AMR techniques^33^.

## Discussion

The use of radiation pressure to induce bead oscillations has two key implications for the basis of PF-OCE. First, the ultra-low radiation-pressure force from a low-NA beam results in picometer-to-nanometer bead oscillation amplitudes, which pushes the limit of interferometric axial displacement sensitivity of phase-sensitive OCT. Our results demonstrate an oscillation amplitude measurement sensitivity of ≤105 pm (for OCT SNR ≥25 dB), which, in practice, corresponds to the smallest detectable oscillation amplitude of 150 pm for a detection threshold of 3 dB. The current sensitivity was achieved after optimization of hardware synchronization and acquisition scheme to minimize galvanometer motion instability.

Second, the use of radiation pressure from a low-NA beam in aqueous media is accompanied by absorption-mediated thermal effects, which can produce responses that may be an order of magnitude larger than the mechanical response induced by radiation-pressure force (Fig. 1a–d). Employing a differential scattering approach with 3- and 0.1-µm beads, our experiments in agarose hydrogels demonstrate that PF-OCE is able to isolate bead mechanical responses induced by modulated radiation pressure by compensating for the photothermal responses of the surrounding hydrogels. The measured total response from the 3-µm beads did not directly correspond to either the mechanical response or the photothermal response alone. These results confirm the importance of accounting for both scattering-mediated radiation pressure and absorption-mediated photothermal responses when using 976-nm photonic excitation from a low-NA beam in aqueous media.

One potential area of application of PF-OCE is the study of dynamic cell–ECM biophysical interactions in 3D environments. Particularly, the embedded scattering beads used in PF-OCE may also readily serve as the fiducial beads in TFM^45^. Recent innovations in TFM strive to extend traditional quasi-static and 2D dynamic studies to 3D volumetric measurements of dynamic cell–ECM interactions, including collective (emergent) cellular behaviors^22,27,28,45^. Previous work on mechanical characterization of hydrogels typically used as ECM in mechanobiology studies reveals drastic variations in the pore shapes and sizes of fibrin hydrogels^60^, and as a result, their local mechanical properties can vary by over an order of magnitude^32,33^. An important capability that would enable such studies is the characterization of spatio-temporal variations in the mechanical properties of hydrogel substrates at subcellular length scales over a millimeter-scale volume at timescales of minutes to hours. OT-AMR is currently still the leading technique for characterization of ECM mechanical properties during live-cell-imaging studies^33^. However, a practical challenge that persists in current OT-AMR studies is the precise alignment of the trapping beam to the center of the marker beads to within 0.1 μm, which must be achieved on each individual bead being probed at a given time. This results in a net measurement time of 8 s per bead achieved by recent state-of-the-art OT-AMR studies^33^. Furthermore, the detection of transverse bead displacements (typically on the order of 10^1^–10^2^ nm) in OT-AMR is implemented via a trans-illumination geometry, which limits the sample thickness (and turbidity) that can be imaged. In this respect, PF-OCE has the benefit of epi-illumination, continuous-scanning acquisition that probes multiple beads at various depths in each B-scan, owing to the use of a low-NA beam to exert transversely localized axial radiation-pressure force over an extended depth range.

Moving toward future implementation of PF-OCE in 3D live-cell-imaging studies, the acquisition scheme presented here can be expanded to accommodate larger-scale volumetric acquisition over timescales of minutes to hours. For instance, consider a 3D multi-cellular imaging study over a volumetric FOV of 1 mm × 1 mm × 200 μm (depth range about the PF beam focal plane with viable PF-OCE mechanical response data) with statistical spatial sampling determined by a 15-µm average edge-to-edge bead separation (corresponding to approximately 48 500 beads within the volume). A feasible PF-OCE acquisition scheme may involve acquiring two volumes serially, each consisting of 3000 BM-mode frames per slow-axis position (achieving 190 pm sensitivity instead of the current 105 pm) and a frame rate of 200 Hz over a fast-axis scan range of 500 µm (the largest scan range achievable by our current galvanometer at this frame rate). At the transverse spatial sampling of 1 μm per pixel, the total PF-OCE acquisition time for the entire volume would be 8.3 h, whereas an OT-AMR measurement (at 8 s per bead, assuming that volumetric measurements can be conducted up to 200 µm depth) would take up to 4.5 days. If we were to use a resonant scanner^55^ and acquire the same PF-OCE dataset at the frame rate of 500 Hz over a fast-axis scan range of 1 mm (for an exposure time of 2 µs per A-scan while maintaining the spatial sampling required to ensure sufficient SNR of OCT images), the total acquisition time would reduce to only 100 min. In this case, PF-OCE offers over 60× improvement in volumetric throughput over the current state-of-the-art OT-based microrheology techniques. Future studies will also address quantitative reconstruction of the local complex shear modulus from the measured PF-OCE bead mechanical responses by utilizing OCT-based depth-resolved measurement of radiation-pressure force^44^. Additionally, such studies may warrant bead sizes smaller than the 3-µm ones used here; the NA of the PF forcing beam could be appropriately increased to optimize forces and the corresponding displacements (Fig. 1b, c). Based on our differential scattering approach, there is also a future possibility of completely endogenous PF-OCE (that is, without embedded beads) that relies on native scattering of different constituents in the biological sample. With the potential to reconstruct microscale viscoelastic properties of hydrogels from variations in complex mechanical responses of embedded beads over millimeter-scale volumes, PF-OCE may unlock new research directions in cell mechanics and mechanobiology. A potential example of this is the study of how micro-mechanical properties and biophysical cell–ECM interactions impact collective behavior^27–29^, such as the emergent 3D migration patterns of invasive cancer cells.

## Methods

### Sample preparation

First, a sample chamber shown in Fig. 2a was fabricated from microscope glass coverslips (Electron Microscopy Sciences, 22 × 22 mm, #0) for each agarose hydrogel sample. The coverslips were bonded together by an RTV silicone adhesive (Permatex, 80050) as shown in Fig. 2a. The chamber was left to cure for 24 h before use.

The five agarose hydrogel concentrations were made by mixing solid agarose polymer (Fisher Scientific, BP1423) with room-temperature distilled water at the concentrations of 0.2, 0.3, 0.4, 0.5, and 1% (w/w). The mixture was repeatedly heated in a microwave oven for 5 s then stirred for 10 s until all visible agarose solid had dissolved and the mixture became clear. Throughout the process, the total weight of the mixture was constantly checked for any loss of water to evaporation; distilled water was added accordingly. Three-micrometer polystyrene microsphere suspension (Sigma-Aldrich, LB30) was added to the dissolved mixture at the concentration of 6 μL mL^−1^ to achieve 15-μm mean edge-to-edge particle spacing. Then, 0.1-μm polystyrene microsphere suspension (Sigma-Aldrich, LB1) was added at a concentration of 0.12 μL mL^−1^ to achieve 2-μm mean edge-to-edge particle spacing. The mixture was stirred by hand to ensure all particles were dispersed before injecting into the pre-made glass chamber. The individual samples were injected such that the sample chamber was filled completely with no air gaps/bubbles. For the side-by-side samples, the sample chamber was partially injected with 1% agarose hydrogel such that it filled one-half of the chamber without any gaps and left to set. Later, the remaining volume in the chamber was filled with 0.2% agarose hydrogel. Finally, the glass chamber was sealed on both opening ends by a liquid sealing glue (Bob Smith Industries, Insta-Cure+). We found that it was crucial to carefully seal the open ends of the chamber to produce a confined container. When the open ends of the glass chamber were not sealed, the hydrogels underwent drastic structural and compositional change due to evaporation of water and the motion of the beads was apparent under the OCT system during data acquisition.

Separate hydrogel samples were made in aluminum trays (The Lab Depot, TLDD43–100) from the same agarose–distilled water mixture for rheometer testing. The samples for the rheometer testing were cut into a disk with diameter of 40 mm and thickness of 2 mm.

### Optical setup

The optical setup (Fig. 1a) for measuring complex OPL oscillation consisted of an SD-OCT system with a broadband superluminescent diode (Thorlabs, LS2000B) with center wavelength of 1300 nm. The OCT beam focused with an NA of 0.14. The transverse and axial resolutions were 4.5 and 3.7 μm in air, respectively (we note that unlike confocal microscopy, OCT does not need high NA to achieve cellular resolution^61^). Combined in free space with the sample arm of the OCT system is a laser diode (RPMC Lasers, R0976SB0500P) at the wavelength of 976 nm (in air) acting as the PF forcing beam. We selected the PF forcing beam wavelength of 976 nm for its low to negligible cytotoxicity reported in previous OTs studies^62^. A BCM was used to optimize the PF forcing beam and ensure that it focused to the same position in 3D space as the OCT beam after going through the same OCT sample arm objective lenses (refer to Supplementary Methods). The waist radius of the PF forcing beam was measured to be 3.19 μm at the focal plane, corresponding to an NA of 0.1 (Supplementary Fig. 1).

### Data acquisition

To maximize the force exerted by the PF forcing beam during acquisition, the alignment between the OCT beam and the PF forcing beam was checked at the beginning of each experiment (Supplementary Methods). A 3D BM-mode acquisition scheme (Fig. 2b) was adopted. For each concentration in the individual samples of 0.2–0.5% agarose hydrogels, each 3D volume consisted of 10 BM-mode datasets, acquired at 10 slow-axis positions along the *y*-axis. Each BM-mode dataset consisted of 6144 frames (200 frames per second) with 256 A-scans per frame (Fig. 2c). This acquisition scheme provides a transverse FOV of 200 μm × 10 μm at the spatial sampling density of 0.8 μm per pixel along the fast *x*-axis and 1 μm per pixel along the slow *y*-axis. Each spatial voxel contains 6144 measurements of the sample response over time. During the acquisition of each BM-mode dataset, the PF forcing beam power was modulated by a function generator (Tektronix, AFG3051C), which sent a continuous 20-Hz sinusoidal modulation waveform to the laser diode controller. Since the modulation was provided externally by a function generator, asynchronous to the OCT acquisition control, we measured the function generator output at the *m*th A-scan in each frame to reconstruct the full PF drive waveform. The function generator voltage at the *m*th A-scan reflected the real part of the complex drive waveform, $\tilde V\left( t \right)$, at that A-scan. From this measurement, we calculated the phase of the drive waveform at the first acquired A-scan from *φ*_drive_ = *φ*_*m*_ − *ωt*_*m*_, where *φ*_*m*_ and *t*_*m*_ denote the phase and time at the *m*th A-scan, respectively. Then, we reconstructed the complex drive waveform as $\tilde V(t) = V_0\exp (i(\omega t + \varphi _{{\mathrm{drive}}}))$, where *V*_0_ denotes the modulation amplitude. At the frame rate of 200 Hz, the OPL oscillation due to the 20-Hz modulation was sampled at 10 distinct phases per modulation cycle. Three such volumes were acquired at different imaging locations in each sample. All synchronization and instrument controls were accomplished via a custom LabVIEW software.

The side-by-side sample was acquired with 208 A-scans spanning a fast-axis range of 208 μm at 490 frames per second and 3072 frames per slow-axis location. Acquiring 125 such slices at 1-μm spacing yielded a 200 μm × 125 μm transverse FOV. Furthermore, the 20-Hz waveform for modulating the PF forcing beam was generated internally via the LabVIEW software (rather than the function generator) in order to ensure precise synchronization with OCT A-scans.

### Data processing

For the individual (single-concentration) samples of agarose hydrogels, all the data processing was implemented in MATLAB. The spatial-domain OCT image was reconstructed with standard procedures (background subtraction, spectrum resampling, dispersion correction, and inverse Fourier transformation). In order to efficiently process large 3D BM-mode datasets, only the depths containing the sample (601 pixels in depth out of 2048 acquired) were reconstructed; this was implemented with a high-speed SD-OCT processing method for depth-selective reconstruction^45,63^. The reconstructed spatial-domain complex OCT image was first segmented into a photothermal data region (corresponding to the 0.1-µm beads and bottom glass surface of the sample chamber) and a total response data region (corresponding to the 3-µm beads) via thresholds based on magnitude of the reconstructed OCT image and OCT SNR (Fig. 3). The thresholds used to generate the results here are defined in Supplementary Table 2. Unless stated otherwise, all remaining processing steps outlined in this section were performed independently on each spatial pixel in the 3D OCT image that passed the thresholds, which encoded OPL oscillation resulting from 20-Hz modulation of the PF forcing beam power.

The OPL oscillations due to the modulated PF forcing beam were estimated with a previously described method to reconstruct complex sample displacement in phase-sensitive OCE^64^. Briefly, the complex phase differences were calculated between every adjacent BM-mode frame at each spatial pixel. The complex phase differences, expressed as *e*^*i*Δ*Φ*(**r**,*t*)^, were first registered to that of the top glass surface of the sample chamber to remove systematic noise and phase drifts across BM-mode frames, then filtered by a median filter (3 × 3 kernel, applied separately to the real and imaginary parts of *e*^*i*Δ*Φ*(**r**,*t*)^). The real-valued phase differences, Δ*Φ*(**r**,*t*), were obtained from the phase angle of *e*^*i*Δ*Φ*(**r**,*t*)^, then, filtered by a Butterworth bandpass filter (±1 Hz pass-band centered around 20 Hz). The complex OPL oscillation at each spatial pixel was obtained after cumulative summation (integration in time) and Hilbert transformation of Δ*Φ*(**r**,*t*) along the time axis. This complex OPL oscillation corresponded to the raw photothermal response, ΔOPL_PT_ (*x*, *y*, *z*, *t*) and total response, ΔOPL_tot_ (*x*_b_, *y*_b_, *z*_b_, *t*), for spatial pixels in the photothermal response data region and total response data region, respectively. The vector (*x*_b_, *y*_b_, *z*_b_) refers the set of pixel coordinates of each spatial pixel comprising the segmented 3-µm bead regions. Note that we omit the argument *ω* included in Eqs. (5)–(7) because *ω* = 2π (20 Hz) is implied for all PF-OCE measurements.

The amplitude and phase-shift w.r.t. *φ*_drive_ of the raw ΔOPL_PT_ (*x*, *y*, *z*, *t*) in each 3D dataset were curve-fit as a function of depth by the theoretical curve obtained from simulation and by a cubic polynomial function, respectively. A cubic polynomial function was used to fit the phase data because the theoretical simulation did not to reproduce the non-zero and depth-dependent phase delay observed experimentally (Supplementary Figs 4 and 5). The curve-fitting was done by minimizing the weighted sum-square error (SSE). To accommodate for low-SNR data with large phase noise, the SSE calculation was weighted by the OCT SNR in each spatial pixel such that measurements with higher SNR were weighted more heavily. The weights, *W*, were given by

8 $$W = \left\{ {\begin{array}{*{20}{l}} 0 \hfill & ; \hfill & {\mathrm{SNR} < 3} \hfill \\ {\frac{{\mathrm{SNR} - 3}}{4}} \hfill & ; \hfill & {3 \le{\mathrm{SNR}}< 7} \hfill \\ 1 \hfill & ; \hfill & {\mathrm{SNR}\ge 7} \hfill \end{array}} \right..$$

For each 3D dataset, the best-fit curves for the amplitude and phase data yielded *A*_PT_ (*z*) and *φ*_PT_ (*z*), respectively. Then, the depth-dependent complex photothermal response, ΔOPL_PT_ (*z*, *t*), was obtained from ${\mathrm{\Delta OPL}}_{{\mathrm{PT}}}(z,t) = A_{{\mathrm{PT}}}(z)e^{i(\omega t + \varphi _{{\mathrm{drive}}} + \varphi _{{\mathrm{PT}}}(z))}$.

The complex mechanical response at each spatial pixel that comprised the segmented 3-µm bead regions was obtained from ΔOPL_mech_ (*x*_b_, *y*_b_, *z*_b_, *t*) = ΔOPL_tot_ (*x*_b_, *y*_b_, *z*_b_, *t*) − ΔOPL_PT_ (*z*_b_, *t*). The isolated ΔOPL_mech_ (*x*_b_, *y*_b_, *z*_b_, *t*) was further filtered, via multiplication by a brick-wall filter (±0.2 Hz pass-band) in the frequency domain, before its amplitude *A*_mech_ (*x*_b_, *y*_b_, *z*_b_) and phase *φ*_mech_ (*x*_b_, *y*_b_, *z*_b_) were extracted. In order to obtain *A*_mech_, *φ*_mech_, *A*_PT_, *φ*_PT_, *A*_tot_, and *φ*_tot_ for each of the 3-µm beads, the spatial pixels that belonged to a given 3-µm bead were identified and grouped together by their pixel coordinates (*x*_b_, *y*_b_, *z*_b_). Then, the median values of *A*_mech_, *φ*_mech_, *A*_PT_, *φ*_PT_, *A*_tot_, and *φ*_tot_ were calculated for each group, yielding the responses of each 3-µm bead. These median values were used to generate the maps of *A*_mech_ and *φ*_mech_ (Fig. 4b, c).

For the side-by-side samples, all data was processed using the same procedure as described above, except for the curving-fitting step to estimate ΔOPL_PT_ (*z*, *t*), which was performed over eight sub-volumes, centered at different positions in the *xy* plane. Six peripheral sub-volumes had transverse dimensions of 100 µm × 25 µm, and two central sub-volumes had transverse dimensions of 100 µm × 50 µm. All side-by-side data processing, except for the photothermal curve-fitting and the final step to estimate the median values of the responses from 3-µm beads, was performed on a NVIDIA Titan Xp GPU with custom C++ software using the CUDA v8.0 Toolkit. This was a key step for practical implementation of PF-OCE for volumetric datasets that resulted in a processing time of 90 min, a 40× improvement over its equivalent in MATLAB.

### Statistical analysis

All statistical analysis was implemented in MATLAB. Two statistical tests were performed. First, a Wilcoxon-type non-parametric test for ordered groups, proposed by Cuzick^65^, was implemented to test the null hypothesis that that there was no statistically significant trend across the four agarose concentrations (that is, the responses from the four samples were not ordered) against the alternative hypothesis that there was a statistically significant trend. The normalized test statistics, *z*_C_, and the associated two-sided *p*-value, *p*_C_, are reported. The data were ordered such that a *z*_C_ > 0 indicates an increasing trend while a *z*_C_ < 0 indicates a decreasing trend. We considered a trend to be statistically significant if *p*_C_ < 0.05. Second, a multiple comparison based on Wilcoxon-type non-parametric Kruskal–Wallis test of variance was implemented to determine if there were statistically significant differences between measurements from any two agarose concentrations. The reported *p*-values reflect the significance of chi-squared (*χ*^2^) statistics on the group-adjusted (Bonferroni correction for multiple comparisons among groups) two-sided pairwise comparison between two agarose concentrations. In both tests, rank-based non-parametric methods were chosen to accommodate for deviation from a normal distribution (Anderson–Darling test for normality) and unequal variances (Barlett’s test for equal variances) among measurements in different agarose concentrations.

Although the variations in the local mechanical responses within a sample can be attributed to the heterogeneity of agarose hydrogels (see Supplementary Discussions for possible sources of variability within a sample), in order to compare these responses to the bulk mechanical properties measured via rheometry and to observe the overall trend of the mechanical response across different concentrations, the reconstructed data (that is, *A*_tot_ (*x*_b_, *y*_b_, *z*_b_), *φ*_tot_ (*x*_b_, *y*_b_, *z*_b_), *A*_mech_ (*x*_b_, *y*_b_, *z*_b_), *φ*_mech_ (*x*_b_, *y*_b_, *z*_b_), *A*_PT_ (*z*_b_), and *φ*_PT_ (*z*_b_)) were subjected to further thresholding to exclude outliers from the responses of each agarose concentration prior to performing the statistical tests. Any spatial pixels that contained *A*_mech_ (*x*_b_, *y*_b_, *z*_b_) values above the 85th percentile or below the 15th percentile of all *A*_mech_ (*x*_b_, *y*_b_, *z*_b_) values in each 3D dataset (that is, the percentiles were calculated separately for each imaged location in each sample), were excluded from the statistical tests. The acceptance or rejection of the null hypothesis by the Cuzick’s test for trend was not affected by this exclusion (Supplementary Fig. 9). The responses of each of the 3-µm beads were then obtained from the median values among all remaining spatial pixels in each 3D dataset that constituted each bead.

All statistical analysis, including all box plots (Fig. 5 and Supplementary Figs 6 and 7), were performed using the median values for each of the 3-µm beads after exclusion of outlier pixels. The total number of beads included in the statistical analysis was *N* = 54, 74, 58, and 52 beads for 0.2%, 0.3%, 0.4%, and 0.5% agarose hydrogel samples, respectively.

### Data availability

All relevant data are available from the authors.

## Electronic supplementary material

Supplementary Information

Peer Review File

Description of Additional Supplementary Files

Supplementary Movie 1

## References

[1] Grier D (2003). A revolution in optical manipulation. Nature.

[2] Stevenson DJ, Gunn-Moore F, Dholakia K (2010). Light forces the pace: optical manipulation for biophotonics. J. Biomed. Opt..

[3] Bowman RW, Padgett MJ (2013). Optical trapping and binding. Rep. Prog. Phys..

[4] Ashkin A, Dziedzic JM, Bjorkholm JE, Chu S (1986). Observation of a single-beam gradient force optical trap for dielectric particles. Opt. Lett..

[5] Block SM, Blair DF, Berg HC (1989). Compliance of bacterial flagella measured with optical tweezers. Nature.

[6] Svoboda K, Schmidt CF, Schnapp BJ, Block SM (1993). Direct observation of kinesin stepping by optical trapping interferometry. Nature.

[7] Molloy JE, Burns JE, Kendrick-Jones J, Tregear RT, White DCS (1995). Movement and force produced by a single myosin head. Nature.

[8] Wang Y (2005). Visualizing the mechanical activation of Src. Nature.

[9] Bustamante C, Bryant Z, Smith SB (2003). Ten years of tension: single-molecule DNA mechanics. Nature.

[10] Forties RA, Wang MD (2014). Discovering the power of single molecules. Cell.

[11] Ashkin A (1970). Acceleration and trapping of particles by radiation pressure. Phys. Rev. Lett..

[12] Guck J (2001). The Optical Stretcher: a novel laser tool to micromanipulate cells. Biophys. J..

[13] Guck J (2005). Optical deformability as an inherent cell marker for testing malignant transformation and metastatic competence. Biophys. J..

[14] Ekpenyong AE (2012). Viscoelastic properties of differentiating blood cells are fate- and function-dependent. PLoS ONE.

[15] Dholakia K, MacDonald MP, Zemanek P, Cizmar T (2007). Cellular and colloidal separation using optical forces. Methods Cell Biol..

[16] Jonas A, Zemanek P (2008). Light at work: the use of optical forces for particle manipulation, sorting, and analysis. Electrophoresis.

[17] Eyckmans J, Boudou T, Yu X, Chen CS (2011). A Hitchhiker’s guide to mechanobiology. Dev. Cell.

[18] Discher DE, Janmey P, Wang YL (2005). Tissue cells feel and respond to the stiffness of their substrate. Science.

[19] Paszek MJ (2005). Tensional homeostasis and the malignant phenotype. Cancer Cell.

[20] Levental KR (2009). Matrix crosslinking forces tumor progression by enhancing integrin signaling. Cell.

[21] Butcher DT, Alliston T, Weaver VM (2009). A tense situation: forcing tumor progression. Nat. Rev. Cancer.

[22] Wirtz D, Konstantopoulos K, Searson PC (2011). The physics of cancer: the role of physical interactions and mechanical forces in metastasis. Nat. Rev. Cancer.

[23] Guilak F (2009). Control of stem cell fate by physical interactions with the extracellular matrix. Cell Stem Cell.

[24] Gilbert PM (2010). Substrate elasticity regulates skeletal muscle stem cell self-renewal in culture. Science.

[25] Wong VW, Akaishi S, Longaker MT, Gurtner GC (2011). Pushing back: wound mechanotransduction in repair and regeneration. J. Invest. Dermatol..

[26] Mammoto T, Mammoto A, Ingber DE (2013). Mechanobiology and developmental control. Annu. Rev. Cell Dev. Biol..

[27] Trepat X (2009). Physical forces during collective cell migration. Nat. Phys..

[28] Tambe D (2011). Collective cell guidance by cooperative intercellular forces. Nat. Mater..

[29] Roca-Cusachs P, Conte V, Trepat X (2017). Quantifying forces in cell biology. Nat. Cell Biol..

[30] Pampaloni F, Reynaud EG, Stelzer EHK (2007). The third dimension bridges the gap between cell culture and live tissue. Nat. Rev. Mol. Cell Biol..

[31] Legant WR (2010). Measurement of mechanical tractions exerted by cells in three-dimensional matrices. Nat. Methods.

[32] Kotlarchyk MA (2011). Concentration independent modulation of local micromechanics in a fibrin gel. PLoS ONE.

[33] Keating M, Kurup A, Alvarez-Elizondo M, Levine AJ, Botvinick E (2017). Spatial distribution of pericellular stiffness in natural extracellular matrices are dependent on cell-mediated proteolysis and contractility. Acta Biomater..

[34] Mejean CO, Schaefer AW, Millman EA, Forscher P, Dufresne ER (2009). Multiplexed force measurements on live cells with holographic optical tweezers. Opt. Express.

[35] Schwingel M, Bastmeyer M (2013). Force mapping during the formation and maturation of cell adhesion sites with multiple optical tweezers. PLoS ONE.

[36] Curtis JE, Koss BA, Grier DG (2002). Dynamic holographic optical tweezers. Opt. Commun..

[37] Scarcelli G, Yun SH (2008). Confocal Brillouin microscopy for three-dimensional mechanical imaging. Nat. Photon..

[38] Scarcelli G (2015). Noncontact three-dimensional mapping of intracellular hydromechanical properties by Brillouin microscopy. Nat. Methods.

[39] Mulligan JA, Untracht GR, Chandrasekaran SN, Brown CN, Adie SG (2016). Emerging approaches for high-resolution imaging of tissue biomechanics with optical coherence elastography. IEEE J. Sel. Top. Quantum Electron..

[40] Kennedy BF, Wijesinghe P, Sampson DD (2017). The emergence of optical elastography in biomedicine. Nat. Photon..

[41] Larin KV, Sampson DD (2017). Optical coherence elastography—OCT at work in tissue biomechanics. Biomed. Opt. Express.

[42] Nichols EF, Hull GF (1901). A preliminary communication of the pressure of heat and light. Phys. Rev..

[43] Ma D, Garrett JL, Munday JN (2015). Quantitative measurement of radiation pressure on a microcantilever in ambient environment. Appl. Phys. Lett..

[44] Leartprapun N, Iyer RR, Adie SG (2018). Depth-resolved measurement of optical radiation-pressure forces with optical coherence tomography. Opt. Express.

[45] Mulligan JA, Bordeleau F, Reinhart-King CA, Adie SG (2017). Measurement of dynamic cell-induced 3D displacement fields *in vitro* for traction force optical coherence microscopy. Biomed. Opt. Express.

[46] Oestreicher HL (1951). Field and impedance of an oscillating sphere in a viscoelastic medium with an application to biophysics. J. Acous. Soc. Am..

[47] Wang LV, Hu S (2012). Photoacoustic tomography: in vivo imaging from organelles to organs. Science.

[48] Li C (2014). Laser induced surface acoustic wave combined with phase sensitive optical coherence tomography for superficial tissue characterization: a solution for practical application. Biomed. Opt. Express.

[49] Skala MC, Crow MJ, Wax A, Izatt JA (2008). Photothermal optical coherence tomography of epidermal growth factor receptor in live cells using immunotargeted gold nanospheres. Nano Lett..

[50] Lapierre-Landry M, Tucker-Schwartz JM, Skala MC (2016). Depth-resolved analytical model and correction algorithm for photothermal optical coherence tomography. Biomed. Opt. Express.

[51] Ren KF, Grehan G, Gouesbet G (1996). Prediction of reverse radiation pressure by generalized Lorenz-Mie theory. Appl. Opt..

[52] Oyen ML (2014). Mechanical characterization of hydrogel materials. Int. Mater. Rev..

[53] Park BH (2005). Real-time fiber-based multi-functional spectral-domain optical coherence tomography at 1.3 um. Opt. Express.

[54] Chang EW, Kobler JB, Yun SH (2012). Subnanometer optical coherence tomographic vibrography. Opt. Lett..

[55] Klein T, Huber R (2017). High-speed OCT light sources and system. Biomed. Opt. Express.

[56] Hale GM, Querry MR (1973). Optical constants of water in the 200-nm to 200-um wavelength region. Appl. Opt..

[57] Pernodet N, Maaloum M, Tinland B (1997). Pore size of agarose gels by atomic force microscopy. Electrophoresis.

[58] Narayanan J, Xiong JY, Liu XY (2006). Determination of agarose gel pore size: absorbance measurements vis a vis other techniques. J. Phys. Confer. Ser..

[59] Boral S, Saxena A, Bohidar HB (2010). Syneresis in agar hydrogels. Int. J. Biol. Macromolec..

[60] Kotlarchyk MA, Botvinick EL, Putnam AJ (2010). Characterization of hydrogel microstructure using laser tweezers particle tracking and confocal reflection imaging. J. Phys. Condens. Matter.

[61] Swanson EA (1993). In vivo retinal imaging by optical coherence tomography. Opt. Lett..

[62] Liang H (1996). Wavelength dependence of cell cloning efficiency after optical trapping. Biophys. J..

[63] Chelliyil RG, Ralston TS, Marks DL, Boppart SA (2008). High-speed processing architecture for spectral-domain optical coherence microscopy. J. Biomed. Opt..

[64] Adie SG (2010). Spectroscopic optical coherence elastography. Opt. Express.

[65] Cuzick J (1985). A Wilcoxon-type test for trend. Stat. Med..

