# Supplementary material for: Photonic force optical coherence elastography for three-dimensional mechanical microscopy
Source: Nat Commun. 2018 May 25;9:2079. doi: 10.1038/s41467-018-04357-8 (PMC5970204; doi:10.1038/s41467-018-04357-8)
Supplement: Supplementary file 1 — Supplementary Information [file 41467_2018_4357_MOESM1_ESM.pdf]

Supplementary information for "Photonic force optical coherence elastography for three-dimensional mechanical microscopy"

Nichaluk Leartprapun[1], Rishyashring R. Iyer[1], Gavrielle R. Untracht[1,a], Jeffrey A. Mulligan[2], and Steven G. Adie[1,*]

[1] Meinig School of Biomedical Engineering, Cornell University, Ithaca, New York 14853

[2] School of Electrical and Computer Engineering, Cornell University, Ithaca, New York 14853

[a] Current affiliation: Optical and Biomedical Engineering Laboratory, School of Electrical, Electronic and Computer Engineering, The University of Western Australia, Perth, Western Australia 6009

* Corresponding author: sga42@cornell.edu

Supplementary Figure 1

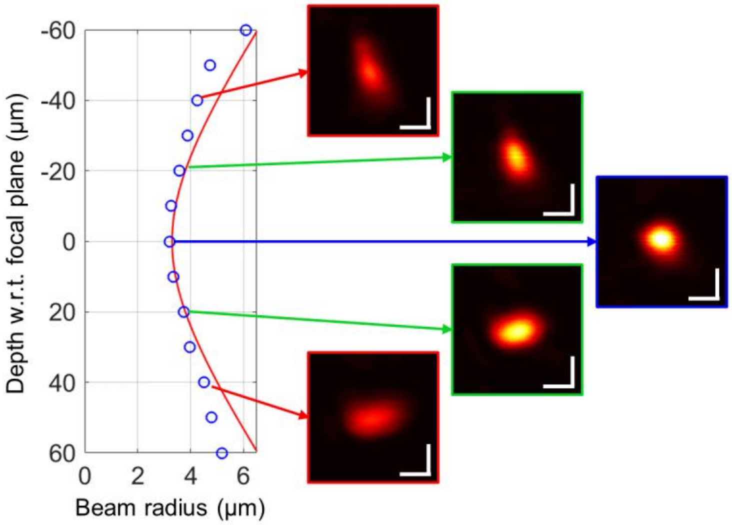

Supplementary Figure 1 legend. Point spread function of the Photonic Force (PF) forcing beam. Plot shows comparison between the $1/e^2$ radii of the Point Spread Function (PSF) obtained from the reflected confocal response of a single 0.5-µm polystyrene bead (**o**) and from the theoretical Gaussian beam profile with the same waist radius (**—**) as function of depth. Images show the *en face* views of the PSF measured at selected depths. Scale bar: 5 µm.

Supplementary Figure 2

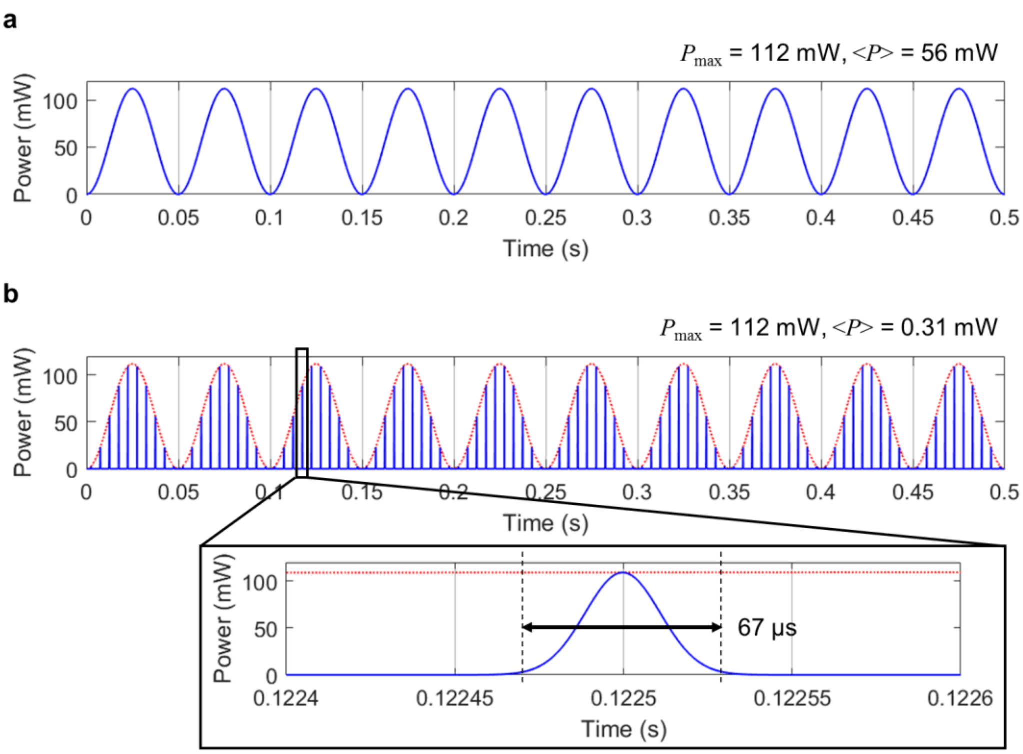

Supplementary Figure 2 legend. Comparison of continuous harmonic modulation versus pulse-train PF drive waveform. a, Continuous 20-Hz sinusoidal drive waveform with peak power, P_{max} , of 112 mW generated by the function generator. **b**, Actual drive waveform felt by each of the 3-μm beads (—) due to beam-scanning along the fast axis in the BM-mode acquisition scheme resembled a pulse-train excitation with a 20-Hz sinusoidal envelope (····). This type of excitation resulted in 3 orders of magnitude lower time-average power, $\langle P \rangle$, compared to the continuous excitation case. The zoomed-up panel shows the pulse width of each pulse excitation based on the dwell time of the PF forcing beam on each 3-μm bead.

Supplementary Figure 3

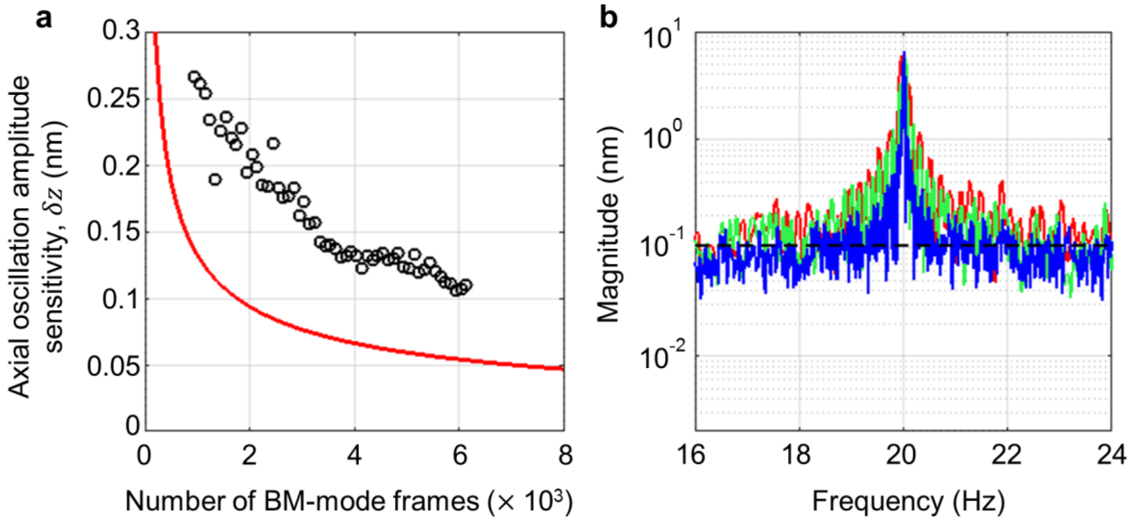

Supplementary Figure 3 legend. Dependence of optical coherence tomography (OCT) axial oscillation amplitude sensitivity on the number of BM-mode frames. a, Observed (**o**) and shot-noise limited theoretical (—) axial oscillation amplitude sensitivity for an OCT signal with SNR of 25 dB as a function of the number of BM-mode frames. The observed oscillation amplitude noise floor with 6,144 BM-mode frames was 105 pm while the theoretical shot-noise limit was 54 pm. **b**, Power spectrum of measured $\Delta \mathrm{OPL}_{\mathrm{tot}}$, truncated to 6,144 (—), 4,044 (—) and 2,044 (—) frames. Dotted line indicates the 105-pm oscillation amplitude sensitivity. We note that, in practice, the smallest detectable oscillation amplitude is approximately 150 pm (3 dB above the noise floor).

Supplementary Figure 4

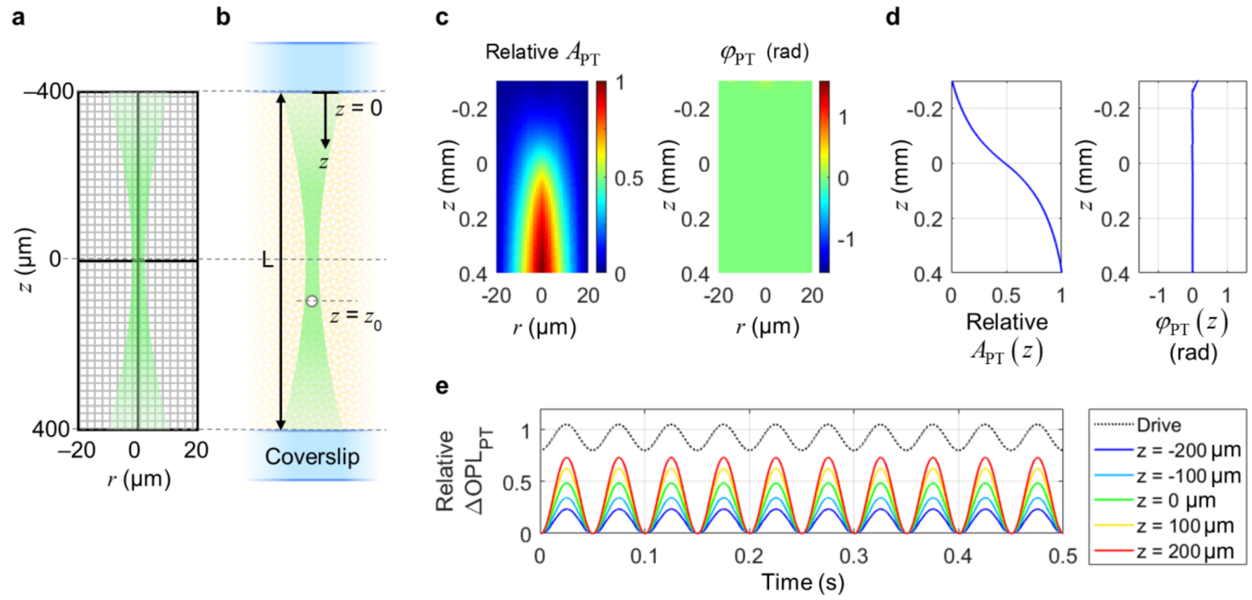

Supplementary Figure 4 legend. Illustrations of geometries used in theoretical simulation of photothermal response and representative simulation output. a, Space-domain in cylindrical coordinate, assuming symmetry about $r=0$ axis, for numerical integration of the heat transfer equation (Supplementary Equations 1-2). **b**, Geometry and dimensions used for cumulative $\Delta\mathrm{OPL}_{\mathrm{PT}}$ calculation by our modified version of Lapierre's model (Supplementary Equations 3-5). **c**, Maps of relative A_{PT} and φ_{PT} from the simulation using a beam with $\lambda = 976$ nm and $w_0 = 3.19$ μm showed spatially varying amplitude but uniformly zero phase delay. **d**, Depth-dependent curves taken at $r=0$ for relative $A_{\mathrm{PT}}(z)$ and $\varphi_{\mathrm{PT}}(z)$. **e**, Time profile of the real part of relative $\Delta\mathrm{OPL}_{\mathrm{PT}}$ taken from various depths at $r=0$. Response at all depths appeared to be in-phase with the drive waveform.

Supplementary Figure 5

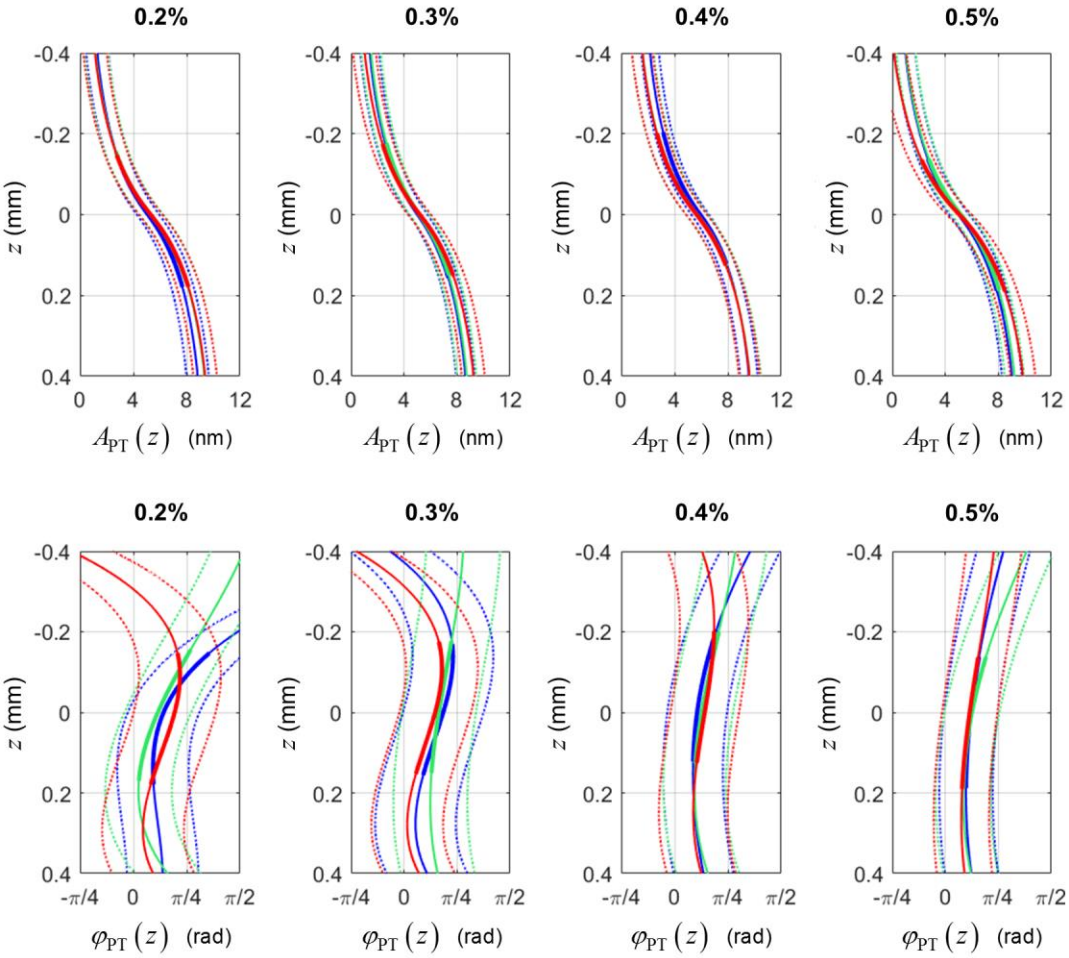

Supplementary Figure 5 legend. Experimental curve fits for the depth-dependent photothermal response. Amplitude (top panels) and phase delay (bottom panels) of depth-dependent photothermal responses obtained from three imaging locations (red, green, and blue lines) in each sample. Bolded lines indicate depth range around focal plane where PF data regions are located. —: best-fitted lines. ·····: ±1 standard deviations. Although the amplitude curves were consistent across different sample concentrations and imaging locations, the phase delay curves were more variable with larger relative uncertainties. Particularly, the discrepancies across the three imaging locations within the same sample appeared to be more prominent at lower agarose concentrations. This could be a result of a larger degree of syneresis (dynamic fluid flow through the agarose polymer matrix, causing structural change due to gel swelling-deswelling over time) at lower agarose concentrations[1-3] as well as larger contributions of apparent photothermal response due to diffusive motions of the 0.1-μm beads in hydrogels with larger pores[4].

Supplementary Figure 6

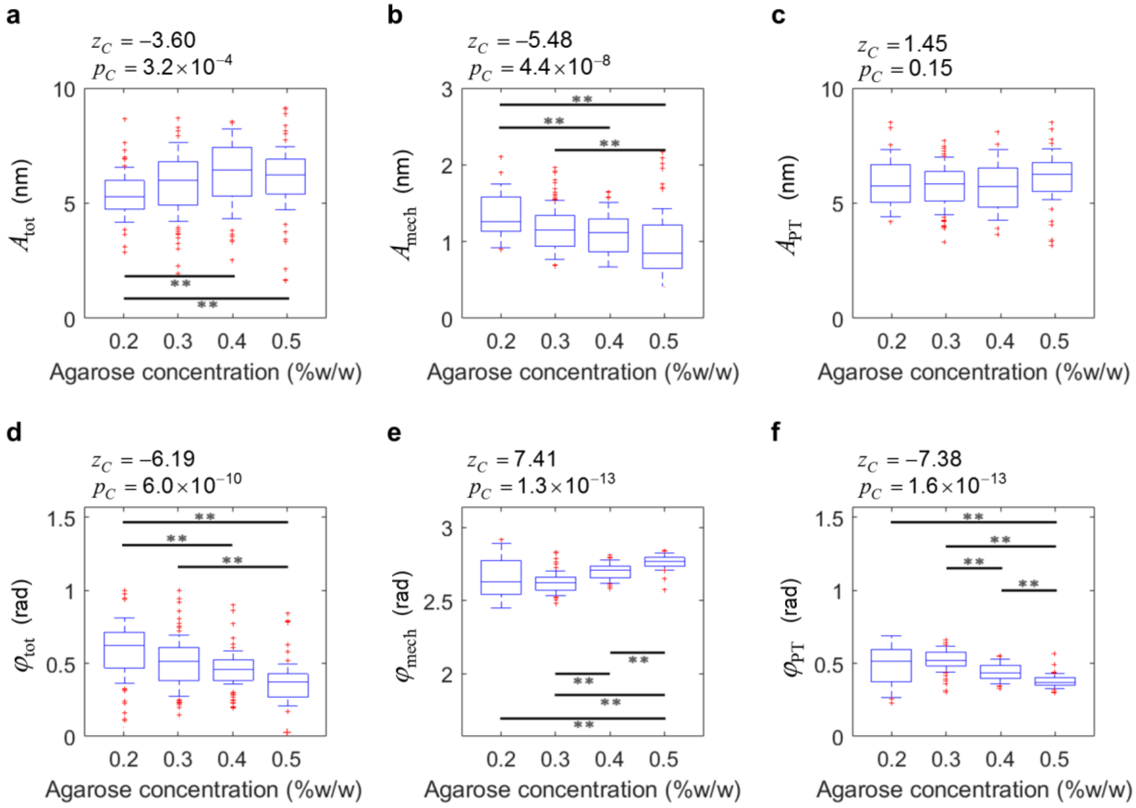

Supplementary Figure 6 legend. Box plots comparing complex total, mechanical and photothermal responses in agarose hydrogels. Magnitude and phase of **a**, **d**, total response (omitted in Fig. 5), **b**, **e**, mechanical response, and **c**, **f**, photothermal response measured by PF-OCE. Horizontal lines within boxes indicate median values, boxes denote interquartile ranges. Error bars span one standard deviation; data outside of this range are shown in red markers. Black bar and asterisks indicate a statistically significant difference between two agarose concentrations per Kruskal-Wallis test at $p < 0.05$ (*) and $p < 0.005$ (**) confidence levels. z_C and p_C , respectively, denote normalized test statistic and associated p-value for Cuzick's test for trend across the four agarose concentrations, ordered from 0.2% to 0.5% w/w; thus, $z_C > 0$ indicates an increasing trend while $z_C < 0$ indicates a decreasing trend. A trend was considered statistically significant if $p_C < 0.05$. Refer to Methods for details of the statistical analysis.

Supplementary Figure 7

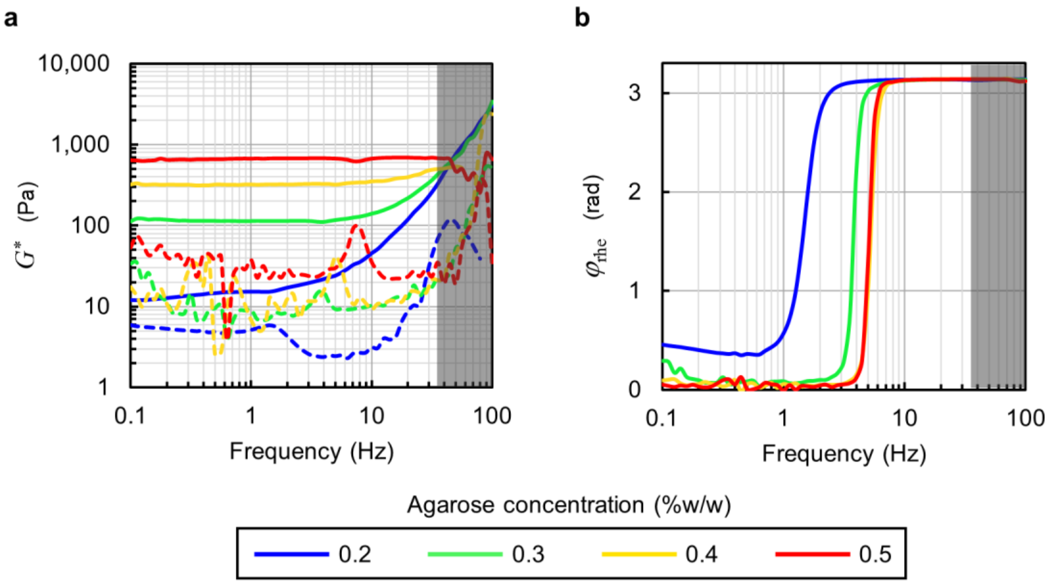

Supplementary Figure 7. Complex shear modulus, G^*, and phase delay, φ_{rhe}, measured by oscillatory test on a parallel-plate shear rheometer. a, Storage moduli (**—**) and loss moduli (**---**) as a function of oscillation frequency. **b**, Raw phase delays between displacement and applied torque. The frequencies at which the sharp rise in phase delays occur roughly correspond to the damped natural frequencies of the samples. Shaded regions correspond to the frequency range over which measurements were unreliable.

Supplementary Figure 8

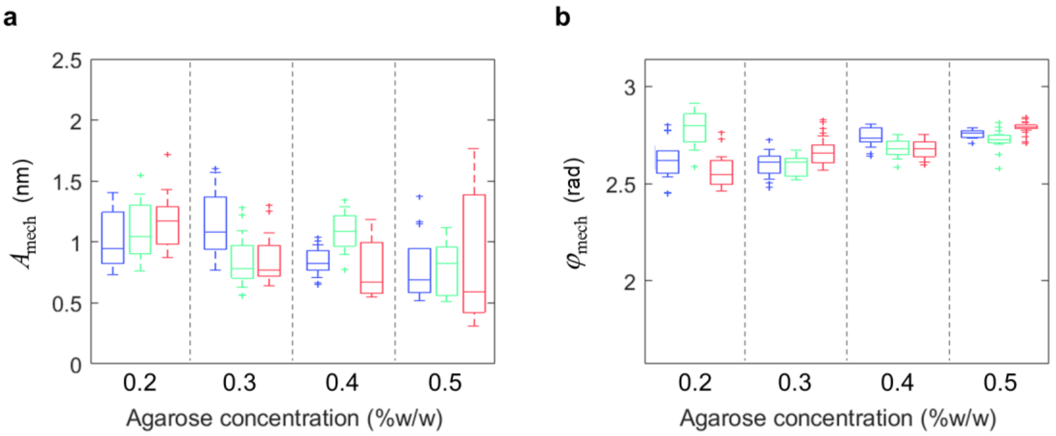

Supplementary Figure 8 legend. Comparison of bead mechanical responses from three imaging locations in each hydrogel sample. Box plots of **a**, amplitude and **b**, phase of bead mechanical responses measured from three imaging locations (indicated by blue, green, and red colors) in each sample. Horizontal lines within boxes indicate median values, boxes denote interquartile ranges. Error bars span one standard deviation; data outside of this range are shown in red markers

Supplementary Figure 9

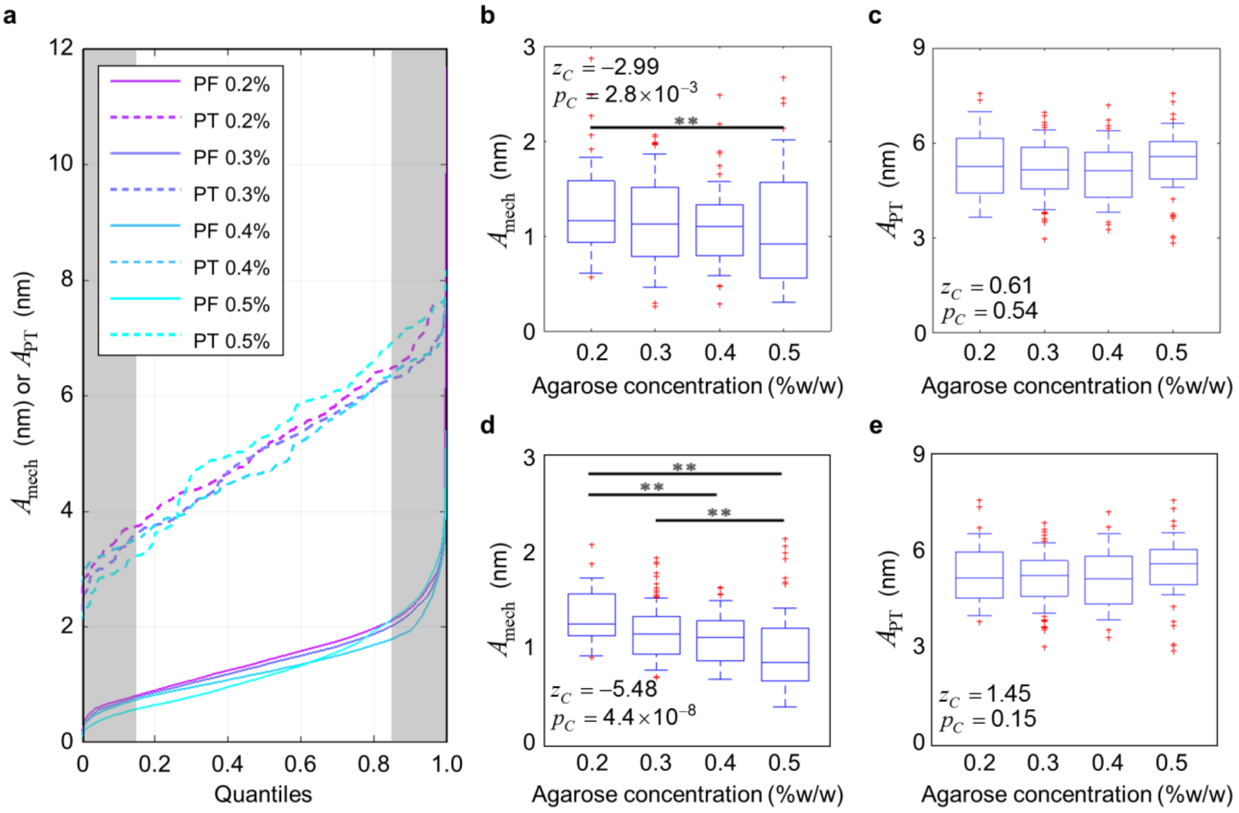

Supplementary Figure 9 legend. Exclusion of outliers for multiple comparisons by Kruskal-Wallis test among four agarose concentrations. a, Quantiles of magnitude of mechanical and photothermal responses measured from each agarose concentration. The dramatic rise in $A_{mech}(x_b, y_b, z_b)$ around the lower and the higher quantiles (shaded regions) may reflect sources of variability described in Supplementary Discussions, and are considered as outliers for each concentration. However, from 0.15 to 0.85 quantiles, the $A_{mech}(x_b, y_b, z_b)$ curves maintain a monotonically increasing linear regime. In contrast, $A_{PT}(z_b)$ curves do not maintain any particular trend versus concentration throughout the entire distribution. The effect of excluding data in the shaded regions (outliers) is visualized by comparing magnitude of mechanical and photothermal responses **b**, **c**, before against **d**, **e**, after the exclusion of data. The general decreasing trend versus concentration is observed for $A_{mech}(x_b, y_b, z_b)$ in both cases, but the contrast between concentrations is more apparent after excluding the outliers from the analysis. No significant trend or difference between concentrations could be observed for $A_{PT}(z_b)$ in either case. In **b**-**e**, horizontal lines within boxes indicate median values, boxes denote interquartile ranges. Error bars span one standard deviation; data

outside of this range are shown in red markers. Black bar and asterisks indicate a statistically significant difference between two agarose concentrations per Kruskal-Wallis test at $p < 0.05$ (*) and $p < 0.005$ (**) confidence levels. z_C and p_C , respectively, denote normalized test statistic and associated p-value for Cuzick's test for trend across the four agarose concentrations, ordered from 0.2% to 0.5% w/w; thus, $z_C > 0$ indicates an increasing trend while $z_C < 0$ indicates a decreasing trend. A trend was considered statistically significant if $p_C < 0.05$. Refer to Methods for details of the statistical analysis.

Supplementary Figure 10

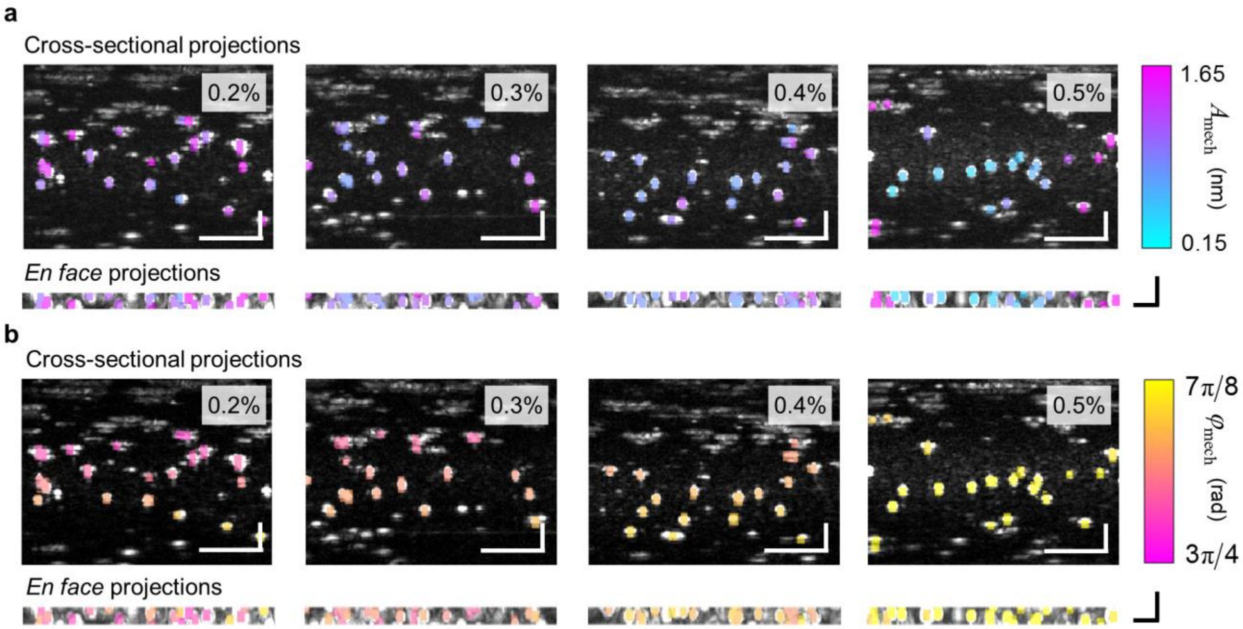

Supplementary Figure 10 legend. Cross-sectional and *en face* projections of complex mechanical response overlaid on the OCT images for agar concentrations. a, $A_{\mathrm{mech}}(x_{\mathrm{b}}, y_{\mathrm{b}}, z_{\mathrm{b}})$ for 0.2-0.5% agar gel w/w **b**, $\varphi_{\mathrm{mech}}(x_{\mathrm{b}}, y_{\mathrm{b}}, z_{\mathrm{b}})$ for 0.2-0.5% agar gel w/w. Scale bars: 50 μm (white, cross-sectional projections) and 20 μm (black, *en face* projections).

Supplementary Table 1. Properties of agarose hydrogels.

**Physical properties**	
Mass density, ρ (kg m^{-3})[5] **	1000
Thermal conductivity, k (W m^{-1} K^{-1})[6] *	0.570 (0.2%)z
	0.554 (0.3%)
	0.538 (0.4%)
	0.523 (0.5%)
Specific heat capacity, c_V (J kg^{-1} K^{-1})[5] **	4187
**Photothermal properties**	
Absorption coefficient, α (m^{-1})[7] **	50 (976 nm)
Thermo-optic coefficient, $\mathrm{d}n/\mathrm{d}T$ (K^{-1})[8] **	–9.17 × 10-5
Thermal expansion coefficient, β (m m^{-1} K^{-1})[9] *	3.68 × 10-6

* Values were obtained from extrapolation of the reported results at higher agarose concentrations.

** Properties not found for agarose hydrogels, values taken for water instead.

Supplementary Table 2. Thresholds for OCT image segmentation.

Notation	Description	Value used *
**Thresholds for 0.1-μm beads in photothermal data region**		
lb_{PT}	Lower bound of reconstructed OCT signal magnitude	700 (a.u.)
ub_{PT}	Upper bound of reconstructed OCT signal magnitude	1400 (a.u.)
$lb_{\mathrm{PT,\ dB}}$	Lower bound of OCT SNR	3 (dB)
$ub_{\mathrm{PT,\ dB}}$	Upper bound of OCT SNR	12 (dB)
**Thresholds for 3-μm beads in total response data region**		
lb_{tot}	Lower bound of reconstructed OCT signal magnitude	5,000 (a.u.)
$lb_{\mathrm{tot,\ dB}}$	Lower bound of OCT SNR	25 (dB)

* Values are specific to the results presented in this manuscript. Values may change according to different acquisition settings or experimental conditions.

Supplementary Methods

Theoretical simulation of photothermal response.

The parameters and the geometry used in the simulation can be found in Supplementary Table 1 and Supplementary Fig. 4, respectively. First, the (three dimensional) 3D heat transfer equation was numerically solved to obtain the change in temperature due to optical absorption by water molecules in the agarose hydrogels. We assumed cylindrical symmetry about the optical axis of the PF forcing beam and assumed zero heat flux normal to each of the four domain boundaries. We also assumed that the PF forcing beam was Gaussian, described by the waist radius obtained from the PSF measurement shown in Supplementary Fig. 1. The differential equation is given by

$$\rho c_V \frac{\partial T}{\partial t} = k\left(\frac{1}{r}\frac{\partial}{\partial r}\left(r\frac{\partial T}{\partial r}\right) + \frac{\partial^2 T}{\partial z^2}\right) + P(r,z,t), \quad (1)$$

and

$$P(r,z,t) = \frac{\alpha P_0}{\pi w^2(z)} e^{-\left(\frac{r^2}{w^2(z)} + \alpha z\right)} e^{i(\omega t + \varphi_{\mathrm{drive}})}, \quad (2)$$

where $r = [-20, 20]$ μm and $z = [-400, 400]$ μm denote the radial and axial coordinates, respectively, T denotes temperature, w denotes beam radius, P_0 denotes beam power and ω denotes the angular modulation frequency. As such, $P(r,z,t)$ denotes the absorbed optical power per unit volume.

After solving for the temperature change, ΔT, the optical path length (OPL) change due to photothermal response, $\Delta\mathrm{OPL}_{\mathrm{PT}}$, was calculated at each time step by

$$\Delta\mathrm{OPL}_{\mathrm{PT}} = \Delta\mathrm{OPL}_{\mathrm{above}} - \Delta\mathrm{OPL}_{\mathrm{below}} \quad (3)$$

where

$$\Delta\mathrm{OPL}_{\mathrm{above}} = \int_0^{z_0}\left(n_0 + \frac{dn}{dT}\Delta T(r,z)\right)\left(1 + \beta\Delta T(r,z)\right)dz - \int_0^{z_0} n_0\, dz, \quad (4)$$

and

$$\Delta\mathrm{OPL}_{\mathrm{below}} = \left[\int_{z_0}^{L} \left(1+\beta\Delta T\left(r,z\right)\right)dz - \int_{z_0}^{L} dz\right]\left(n_0 + \frac{dn}{dT}\Delta T\left(r,z_0\right)\right). \tag{5}$$

The expressions $\Delta\mathrm{OPL}_{\mathrm{above}}$ and $\Delta\mathrm{OPL}_{\mathrm{below}}$ describe the OPL change measured on the bead due to the photothermal response of the portion of the medium located above the bead ($z \le z_0$) and below the bead ($z_0 < z \le L$), respectively. This geometry is illustrated in Supplementary Fig. 4. We note that the model by Lapierre *et al.*[10] did not take into account $\Delta\mathrm{OPL}_{\mathrm{below}}$ and $\Delta\mathrm{OPL}_{\mathrm{PT}}$ was simply given by Supplementary equation (4). The measurements by Lapierre *et al.*[10] were acquired in a phantom with absorbing particles embedded in a less absorbing medium inside a large unconfined container, which allowed the medium surrounding the particles to expand freely due to the heating of the absorbing particles, and the OPL change depended only on the photothermal response of the medium above the bead. In other words, $\Delta\mathrm{OPL}_{\mathrm{PT}} = \Delta\mathrm{OPL}_{\mathrm{above}}$ when the thermal expansion of the medium located below the bead had negligible effect on the physical displacement of the bead relative to the top of the sample ($z = 0$). Here, we included the contribution of both the thermal expansion of the medium above the bead, which pushed the bead away from $z = 0$, in Supplementary equation (4) and the thermal expansion of the medium beneath the bead, which pushed the bead closer to $z = 0$, in Supplementary equation (5) to account for our thin confined sample configuration. In Supplementary equation (5), the terms inside the square brackets describe the physical displacement of the bead due to the thermal expansion of the medium below the bead. To obtain the OPL change, this physical displacement is multiplied by the refractive index of the medium where the bead is located, given by the remaining terms in Supplementary equation (5).

The initial refractive index of the medium, n_0, was assumed to be 1.4. We also used $P_0 = 1\,\mathrm{W}$ in the simulation to generate large enough OPL change to ensure that the numerical solutions remained above the machine precision limit ($\approx 10^{-12}$). As a result, the magnitude of the simulated $\Delta\mathrm{OPL}_{\mathrm{PT}}$ could not be used for direct comparison against the experimentally measured A_{PT}. Alternatively, we normalized

the magnitude of $\Delta\mathrm{OPL}_{\mathrm{PT}}$ by $\left|\Delta\mathrm{OPL}_{\mathrm{PT}}(r,z)\right| / \left|\Delta\mathrm{OPL}_{\mathrm{PT}}(0,L)\right|$ and obtained relative A_{PT}. See Supplementary Fig. 4 for example of simulation output.

Order-of-magnitude estimation of relative force and displacement of 3-μm and 0.1-μm beads.

In order to measure both the total response, $\Delta\mathrm{OPL}_{\mathrm{tot}}$, and the photothermal response, $\Delta\mathrm{OPL}_{\mathrm{PT}}$, we used two bead sizes: higher scattering 3-μm beads provided access to $\Delta\mathrm{OPL}_{\mathrm{tot}}$ and low-scattering 0.1-μm beads allowed us to isolate $\Delta\mathrm{OPL}_{\mathrm{tot}}$. The 3-μm beads were added to achieve a mean particle separation of 15 μm in 3D. The 0.1-μm beads were added at a much higher density, achieving a mean particle separation of only 2 μm. In order to justify our assumption that the OPL oscillation measured on the 0.1-μm beads reflected the photothermal response alone, with negligible contribution of the mechanical response, we considered the theoretical prediction of two extreme cases.

<u>Theoretical prediction (weak mechanical coupling limit).</u> We considered two single-bead scenarios (either 0.1-μm or 3-μm beads), neglecting the presence of multiple beads within the excitation volume. Essentially this assumes that the displacements of a bead are completely independent to those of nearby beads. In this case, the relative magnitude of radiation-pressure force between the 3-μm and the 0.1-μm beads can directly be obtained from separate GLMT simulations for each bead size. We obtained the ratio of radiation-pressure force magnitude per unit power for the two bead sizes of $\bar{F}_{\mathrm{rad,3\mu m}} / \bar{F}_{\mathrm{rad,0.1\mu m}} \approx 10^{5}$. Likewise, the resulting bead oscillation amplitude could be obtained from Oestreicher's model[11], independently for each bead size. We obtained the resulting ratio of bead oscillation amplitude per unit power of $\bar{u}_{0,3\mu m} / \bar{u}_{0,0.1\mu m} \approx 10^{4}$.

<u>Theoretical predication (strong mechanical coupling limit).</u> We considered an equivalent rigid body scenario and accounted for the presence of multiple 0.1-μm beads (or a separate scenario with a single 3-μm bead) within the excitation volume. We approximated the PF excitation volume as a rigid sphere with radius of 3.19 μm, the $1/e^{2}$ radius of our PF forcing beam. In the strong mechanical coupling limit (all beads within the excitation volume are connected by rigid rods), the net external force acting on the rigid PF excitation volume was the sum of all forces on all beads inside the excitation volume. Given our mean particle separation, the ratio of the number of beads inside the excitation volume was $N_{3\mu m} / N_{0.1\mu m} \approx 10^{-2}$. In this case, we obtained the net radiation-pressure force ratio on the PF excitation volume for the two scenarios of $\bar{F}_{\mathrm{rad,3\mu m}} / \bar{F}_{\mathrm{rad,0.1\mu m}} \approx 10^{5} \cdot 10^{-2} = 10^{3}$. Since the excitation volume was a

rigid sphere of the same size for both cases, we also obtained the resulting bead oscillation amplitude ratio of $\bar{u}_{0,3\mu m}/\bar{u}_{0,0.1\mu m} \approx 10^3$.

Experimental prediction based on OCT scattering. We expected that the actual relative difference in the oscillation amplitude of the 3-µm and 0.1-µm beads in our experiments would lie somewhere between these two extreme theoretical cases for weak or strong mechanical coupling. As an experimental comparison to our theoretical estimation, we used the observed OCT scattering intensity to infer the relative magnitude of radiation pressure on the two bead sizes. We note that this assumes that the relative backscattering from the 0.1-µm versus 3-µm beads at 976 nm is comparable to the relative backscattering collected by the OCT system from the 0.1-µm versus 3-µm beads at 1,300 nm. The ratio of observed maximum OCT scattering intensity for the two bead sizes was $\left|\tilde{S}_{3\mu m}\right|^2 / \left|\tilde{S}_{0.1\mu m}\right|^2 \approx 10^3$, where $\tilde{S}$ denotes the complex OCT signal. The OCT scattering intensity in each pixel of the OCT image included the contribution from all beads inside the point spread function (PSF) of the OCT beam. At the focal plane, the OCT beam had comparable waist radius to the PF forcing beam. Given our mean particle separation, the ratio of the number of beads inside the PSF of the OCT beam was $N_{3\mu m}/N_{0.1\mu m} \approx 10^{-2}$. Assuming all beads inside the PSF contributed equally to the observed OCT scattering intensity, we arrived at the radiation-pressure force per one 3-µm bead to radiation-pressure force per one 0.1-µm bead ratio of $\bar{F}_{rad,3\mu m}/\bar{F}_{rad,0.1\mu m} \approx 10^3/10^{-2} = 10^5$, which agrees with our theoretical prediction in the weak mechanical coupling limit.

Characterization of the PF forcing beam.

The point spread function (PSF) of the PF forcing beam was characterized on a single 0.5-μm polystyrene bead on a monolayer phantom in air. A photoreceiver (Newport, 2051-FS) was used to collect the reflected confocal response from a bead as the monolayer phantom was translated to various depths. The monolayer phantom was made by first, diluting a stock solution of 0.5-μm polystyrene bead suspension (Sigma-Aldrich, LB5, 10% solids) in ethanol (VWR, Ethanol, Pure) to a 1:10^9 volume ratio. A 1-μL drop of the diluted microsphere solution was spread on top of an anti-reflection-coated plano-convex lens (Thorlabs, LA1213-B), then left to sit until all solvent had evaporated. The AR-coated plano-convex lens was necessary to sufficiently reduce background reflection detected by the photoreceiver. The PSF of the PF forcing beam used in the experiments in this paper is shown in Supplementary Fig. 1.

Beam alignment procedure.

To maximize the force exerted by the PF forcing beam during the acquisition, the PF forcing beam was aligned to the OCT imaging beam such that the two beams focused to the same position in 3D space. This alignment was checked before every experiment. The position of the PF forcing beam was adjusted by the beam control module (BCM) consisting of six components: two collimating lenses, two spherical-aberration-compensation lenses and two right-angle mirrors. A photoreceiver (Newport, 2051-FS) was used to detect the reflected confocal response of the PF forcing beam. First, the alignment in the axial direction (along the optical axis of the two beams) was done by imaging a flat glass slide. The OCT focal plane was located by translating the glass slide in the axial direction and identifying the depth at which the detected OCT signal from the glass surface was maximized. The PF forcing beam focal plane was similarly located by identifying the depth at which the intensity detected by the photoreceiver was maximized. The position of the collimating lenses in the BCM were adjusted until the focal planes of the two beams were coplanar. Next, the alignment in the transverse plane was determined by imaging a USAF target. The confocal image from the PF forcing beam was compared to the *en face* view of the 3D OCT image. The right-angle mirrors in the BCM were tipped and tilted to steer the PF forcing beam in the transverse plane until both images of the USAF target were aligned.

Calculation of OCT SNR and shot noise-limited oscillation amplitude sensitivity.

The oscillation amplitude sensitivity, fundamentally limited by the OCT phase noise floor, specifies the best achievable precision of the OPL oscillation measurements. In the shot noise limit, the smallest detectable phase difference between two adjacent BM-mode frames (that is, phase difference at a particular spatial pixel at two time points), $\delta\Delta\phi$, depends on the SNR of the OCT signal and is given by Park *et al.*[12]

$$\delta\Delta\phi = \frac{1}{\sqrt{\mathrm{SNR}}}. \tag{6}$$

Then, the physical (not OPL) oscillation amplitude sensitivity, δz, obtained from the reconstructed OPL oscillation (a series of phase difference measurements) is given, based on Chang *et al.*[13], by

$$\delta z = \frac{1}{\sqrt{\mathrm{SNR}\cdot N_\mathrm{A}\cdot N_\mathrm{B}}}\frac{\lambda}{4\pi n_\mathrm{med}}, \tag{7}$$

where n_med denotes refractive index of the medium while N_A and N_B denote the number of samples per modulation cycle and the total number of modulation cycles measured, respectively. We note that the expression from Chang *et al.*[13] differs from Supplementary equation (7) by a factor of $1/\sqrt{2}$ because the authors considered the smallest detectable phase, $\delta\phi = 1/\sqrt{2\cdot\mathrm{SNR}}$, whereas we considered the smallest detectable phase difference between two adjacent BM-mode frames, $\delta\Delta\phi$[12], since this was what we used in our calculation of ΔOPL. For the 6,144-frame BM-mode acquisition scheme implemented in our experiments, N_A was kept at 10 frames per modulation cycle (200-Hz frame rate at 20-Hz modulation frequency) and N_B was 614 full modulation cycles per BM-mode dataset. The SNR was experimentally approximated by

$$\mathrm{SNR}(x,y,z_i) = \left(\frac{\sum_{i-2}^{i+2}\left|\tilde{S}_\mathrm{signal}(x,y,z_i)\right|}{\sum_{i-2}^{i+2}\left|\tilde{S}_\mathrm{noise}(z_i)\right|}\right)^2, \tag{8}$$

where z_i denotes the pixel depth corresponding to the maximum OCT intensity on a particle, $\tilde{S}_\mathrm{signal}(x,y,z)$ denotes the complex OCT signal after reconstruction, and $\tilde{S}_\mathrm{noise}(z)$ denotes the complex

depth-dependent noise floor of the OCT signal. $\tilde{S}_{\mathrm{noise}}(z)$ was obtained from the difference of two reconstructed space-domain OCT background images, averaged across the transverse plane to obtain a depth-dependent complex OCT noise. The theoretical and observed oscillation amplitude sensitivity as a function of the number of BM-mode frames are shown in Supplementary Fig. 2. The observed displacement sensitivity as a function of the number of BM-mode frames was obtained from the Fourier transform of the time-domain $\Delta\mathrm{OPL}_{\mathrm{tot}}$, truncated to a decreasing number of frames from the original 6,144-frame data. The oscillation amplitude noise floor was calculated from the root-mean-square of the noise spectrum adjacent to the 20-Hz response peak.

Parallel-plate shear rheometer measurements.

A parallel-plate shear rheometer (TA Instruments DHR-3) with 40 mm diameter Peltier plate was used to measure the bulk complex shear modulus and phase delay of the agarose hydrogels. 3 samples were tested for each agarose concentration; each sample was tested 3 times consecutively. With these measurement conditions, we note that the rheometer testing does not replicate the boundary conditions presented in the PF-OCE measurements. Each test consisted of an oscillatory sweep from 0.1-100 Hz with torque amplitude of 10 μN·m. The complex shear modulus and phase delay from a representative test for each agarose concentration can be found in Supplementary Fig. 5. Although the shear rheometer could operate up to 100-Hz oscillation frequency, results at higher frequencies (typically greater than 20 Hz) are more prone to errors due to sample slippage and inaccurate phase angle measurements above the damped natural frequency (indicated by the sharp rise in phase delay). Any data points that reported negative values for either the real or the imaginary part of the complex shear modulus were excluded because they indicated inaccurate measurements.

Supplementary Discussion

Reliability and accuracy of the theoretical simulation of the photothermal response.

In principle, the contributions of both amplitude and phase of $\Delta\mathrm{OPL}_{\mathrm{PT}}$ in a homogeneous sample may be obtained from the theoretical simulation based on the model of photothermal phenomena in Photothermal OCT (PT-OCT). However, we were not able to ascertain the accuracy of our theoretical photothermal response simulation under our experimental conditions due to several reasons. Firstly, many of the material properties required for the simulation were not available in the literature for our specific agarose hydrogels. We made approximations by taking properties of water or extrapolated from the available results in the literature to complete our simulation (Supplementary Table 1). Secondly, our current numerical simulation in MATLAB was limited by machine precision, in addition to the fact that we might not have accurately modelled the boundary conditions presented during the experiments. For instance, we were not able to account for the effects of the glass chamber, which could act as a heat sink that may affect the heat transfer process. Thirdly, our simulation might not have accurately accounted for all photothermal phenomena presented in the experiments. Given the similarities between our experimental conditions and those in existing PT-OCT studies, we adapted our model of OPL change due to absorption from PT-OCT literature. We accounted for two photothermal phenomena: the thermo-optic effect and thermal expansion. However, other photothermal phenomena such as photo-elastic effect[14], acousto-optic effects[15] and thermal forces[16,17] could also be present; we did not consider them to be significant photothermal contributions due to the absence of their discussion in PT-OCT literature. Further investigation into these photothermal phenomena will be needed to incorporate them into our simulation. Finally, our current simulation did not reproduce the phase delay we experimentally observed in the measured $\Delta\mathrm{OPL}_{\mathrm{PT}}$ from the 0.1-μm beads (Supplementary Fig. 4). As a result, we could not use the theoretical simulation of $\Delta\mathrm{OPL}_{\mathrm{PT}}$ by itself to isolate the mechanical response. However, since the simulation produced the depth-dependent photothermal response amplitude that agreed with the general trends with observed on the 0.1-μm beads, we were able to use the functional form of the simulated $A_{\mathrm{PT}}(z)$ curve to fit the experimental data.

Sources of variability in PF-OCE measurements.

Factors that may have contributed to the variability in the mechanical responses of the 3-μm beads in each agarose hydrogel sample include noise in OPL oscillation measurements, possible errors in compensating for the photothermal responses (which we have discussed in the main article), the BM-mode beam-scanning acquisition scheme, and microscale mechanical heterogeneity of the agarose hydrogels.

The accuracy of OPL oscillation measurements is dependent on the SNR of the complex OCT signal. This source of error is most prominent in the photothermal response measured from the 0.1-μm beads, which have low OCT SNR (0-12 dB) due to their weak scattering. However, under the assumption of transversely uniform photothermal response, the precision of measuring variations in bead total responses within each sample is governed by the sensitivity of the OPL oscillation measurements on the 3-μm beads, which have higher SNR (>25 dB). Our observed oscillation amplitude sensitivity was approximately 105 pm (Supplementary Fig. 2). Under the assumption of transversely uniform photothermal response (i.e., the error from the photothermal curve fits imposes a depth-dependent systematic error on the isolated mechanical response), this corresponds to the smallest practically detectable differences in A_{mech} of various beads within a sample of 150 pm (3 dB above the noise floor) for any A_{mech} values larger than 0.7 nm (the uncertainty of $A_{\mathrm{PT}}(z)$ curves). In addition to OPL measurement errors and data-acquisition-related factors, Brownian motion of the beads inside porous structures of the agarose hydrogels may also confound the measurements of OPL oscillation induced by the PF forcing beam. It has been shown that Brownian motion of scattering particles resulted in a complex OCT signal with a Lorentzian distribution in the temporal frequency domain, which could also contribute to our observed displacement noise floor being above the shot noise limit.

As a result of the 3D BM-mode acquisition scheme the PF forcing beam was transversely aligned to the centre of each 3-μm bead for only a fraction of the measurements (approximately 10 out of 60 spatial pixels that constituted each bead). In other words, the magnitude of the radiation-pressure force exerted on a given 3-μm bead was varied as the PF forcing beam was scanned over different parts of each bead. Thus, we also expect that the resulting oscillations of the 3-μm beads could also be affected by this variation in the magnitude of force due to the scanning of the PF forcing beam.

Part of the variability observed in the maps of amplitude and phase of the 3-μm bead mechanical responses (Fig. 4b, c in the main manuscript) could also reflect the actual microscale heterogeneity that was present in the agarose hydrogels. Existing evidence of structural and compositional variability in agarose hydrogels support the notion that agarose hydrogels are mechanically heterogeneous at the microscale. Agarose hydrogel is composed of aggregated agar double-helix polymer matrix[18,19], creating a porous structure that holds water within its pores (diameter on the order of 1-6 μm for 0.2-0.5% agarose hydrogels)[4,20]. Additionally, the distribution of pore sizes of one concentration overlaps with that of others based on atomic force microscopy measurements[20]. Furthermore, studies with optical tweezers based active microrheology on fibrin hydrogels show that the viscoelastic modulus of hydrogels can have variations that are over an order of magnitude[21]. The porous structure could result in spatially heterogeneous mechanical responses measured by PF-OCE on each of the 3-μm beads. For instance, beads with larger A_{mech} in each sample may be located inside larger pores, diffusing in the fluid phase of the biphasic hydrogels, whereas those with lower A_{mech} may be trapped in the solid phase made up of agarose polymer matrix. Furthermore, the microstructure of agarose hydrogels is known to change over time due to multiple naturally occurring dynamic processes, including agarose fibre aggregation and local water expulsion from pores, collectively called ‘syneresis’[1-3]. This dynamic change may have caused the variability between measurements from the different imaging locations in each sample (Supplementary Fig. 8), which were acquired at different times (separated by more than 1 hour) after the sample was first made.

Supplementary References

1 Mao, R., Tang, J. & Swanson, B. G. Water holding capacity and microstructure of gellan gels. *Carbohydr. Polym.* **46**, 365-271 (2001).

2 Boral, S., Saxena, A. & Bohidar, H. B. Syneresis in agar hydrogels. *Int. J. Biol. Macromolec.* **46**, 232-236 (2010).

3 Divoux, T., Mao, B. & Snabre, P. Syneresis and delayed detachment in agar plates. *Soft Matter* **11**, 3677-3685 (2015).

4 Narayanan, J., Xiong, J. Y. & Liu, X. Y. Determination of agarose gel pore size: Absorbance measurements vis a vis other techniques. *J Phys Confer Ser* **28**, 83-86 (2006).

5 *M11507; 7732-18-5;* (The Royal Society of Chemistry).

6 Zhang, M. *et al.* Experimental Determination of Thermal Conductivity of Water-Agar Gel at Different Concentrations and Temperatures. *J. Chem. Eng. Data* **56**, 859-864 (2011).

7 Hale, G. M. & Querry, M. R. Optical Constants of Water in the 200-nm to 200-um Wavelength Region. *Appl. Opt.* **12**, 555-563 (1973).

8 Hawkes, J. B. & Astheimer, R. W. The Temperature Coefficient of the Refractive Index of Water. *J. Opt. Soc. Am.* **38**, 804-806 (1948).

9 Aizawa, M. & Suzuki, S. Properties of Water in Macromolecular Gels. III. Dilatometric Studies of the Properties of Water in Macromolecular Gels. *Bull. Chem. Soc. Jpn.* **44**, 2967-2971 (1971).

10 Lapierre-Landry, M., Tucker-Schwartz, J. m. & Skala, M. C. Depth-resolved analytical model and correction algorithm for photothermal optical coherence tomography. *Biomed. Opt. Exp.* **7**, 2607-2622 (2016).

11 Oestreicher, H. L. Field and Impedance of an Oscillating Sphere in a Viscoelastic Medium with an Application to Biophysics. *J. Acous. Soc. Am.* **23**, 707-714 (1951).

12 Park, B. H. *et al.* Real-time fiber-based multi-functional spectral-domain optical coherence tomography at 1.3 um. *Opt. Exp.* **13**, 3931-3944 (2005).

13 Chang, E. W., Kobler, J. B. & Yun, S. H. Subnanometer optical coherence tomographic vibrography. *Opt. Lett.* **37**, 3678-3680 (2012).

14 Wang, L. V. & Hu, S. Photoacoustic Tomography: In Vivo Imaging from Organelles to Organs. *Science* **335**, 1458-1462 (2012).

15 Hajireza, P., Shi, W., Bell, K., Paproski, R. J. & Zemp, R. J. Non-interferometric photoacoustic remote sensing microscopy. *Light Sci. Appl.* **6**, e16278 (2017).

16 Nichols, E. F. & Hull, G. F. A preliminary communication of the pressure of heat and light. *Phys. Rev. (Series I)* **13**, 307-320 (1901).

17 Akhtaruzzaman, A. F. M. & Lin, S. P. Photophoresis of absorbing particles. *J. Colloid Interface Sci.* **61**, 170-182 (1976).

18 Djabourov, M., Clark, A. H., Rowlands, D. W. & Ross-Murphy, S. B. Small-angle x-ray scattering characterization of agarose sols and gels. *Macromolecules* **22**, 180-188 (1989).

19 Zucca, P., Fernandez-Lafuente, R. & Sanjust, E. Agarose and Its Derivatives as Supports for Enzyme Immobilization. *Molecules* **21**, 1577 (2016).

20 Pernodet, N., Maaloum, M. & Tinland, B. Pore size of agarose gels by atomic force microscopy. *Electrophoresis* **18**, 55-58 (1997).

21 Kotlarchyk, M. A. *et al.* Concentration Independent Modulation of Local Micromechanics in a Fibrin Gel. *PLoS ONE* **6**, e20201 (2011).
