# Supplementary material for: Photonic force optical coherence elastography for three-dimensional mechanical microscopy
Source: Nat Commun. 2018 May 25;9:2079. doi: 10.1038/s41467-018-04357-8 (PMC5970204; doi:10.1038/s41467-018-04357-8)
Supplement: Supplementary file 3 — Description of Additional Supplementary Files [file 41467_2018_4357_MOESM3_ESM.pdf]

Description of Additional Supplementary Files

Supplementary Movie 1: A continuous sequence of *en face* and cross-sectional projections of A_{mech} (x_b, y_b, z_b) and A_{PT} (x, y, z) in 2D and 3D space, shown with their corresponding OCT images. Scale bars: 20 µm.
