# Supplementary material for: Photonic force optical coherence elastography for three-dimensional mechanical microscopy
Source: Nat Commun. 2018 May 25;9:2079. doi: 10.1038/s41467-018-04357-8 (PMC5970204; doi:10.1038/s41467-018-04357-8)
Supplement: Supplementary file 2 — Peer Review File [file 41467_2018_4357_MOESM2_ESM.pdf]

Editorial Note: This manuscript has been previously reviewed at another journal that is not operating a transparent peer review scheme. The manuscript was considered suitable for publication without further review at *Nature Communications.*
